# Integrative plasma proteomics and myeloid- interferon profiling reveal an AI-validated vascular- endothelial stress signature distinguishing SLE flare from remission in an Indian cohort a discovery – phase study

**DOI:** 10.3389/fimmu.2026.1819049

**Published:** 2026-06-03

**Authors:** Abhibroto Karmakar, Santanu Mishra, Uma Kumar, Rachana Kamath, Vinod Ravindran, Varashree Bolar Suryakanth, Smitha Prabhu, Shankar Prasad Nagaraju, Mukhyaprana M. Prabhu, Subhradip Karmakar

**Affiliations:** 1 Department of General Medicine, Kasturba Medical College, Manipal Academy of Higher Education, Manipal, India; 2 Department of Biochemistry, All India Institute of Medical Sciences, New Delhi, India; 3 Department of Gastroenterology and Hepatology, Kasturba Medical College, Manipal Academy of Higher Education, Manipal, India; 4 Department of Rheumatology, All India Institute of Medical Sciences, New Delhi, India; 5 Department of Rheumatology, Centre for Rheumatology, Kozhikode, Kerala, India; 6 Department of Biochemistry, Kasturba Medical College, Manipal Academy of Higher Education, Manipal, India; 7 Department of Dermatology, Kasturba Medical College, Manipal Academy Higher Education, Manipal, India; 8 Department of Nephrology, Kasturba Medical College, Manipal Academy of Higher Education, Manipal, India

**Keywords:** biomarker, cytokine, explainable AI (XAI), machine learning, multiomics, precision medicine, proteomics, SLE - systemic lupus erythematosus

## Abstract

**Background:**

Systemic lupus erythematosus (SLE) is a heterogeneous autoimmune disorder characterized by unpredictable flares and variable clinical quiescence. Despite validated clinical indices like the British Isles Lupus Assessment Group (BILAG) score, reliable molecular biomarkers for monitoring disease activity remain limited, particularly in underrepresented South Asian populations. Weaimed to identify arobust molecular framework to distinguish SLE flares from remission in an Indian cohort.

**Methods:**

We conducted a discovery-phase study in an Indian cohort (n=16) stratified by Easy-BILAG scoring. Plasma proteomic profiling via LC-MS/MS was integrated with targeted cytokine quantification using the Olink Proximity Extension Assay (PEA). Differential expression and network analyses delineated immune-regulatory, hypoxic-vascular, and myeloid-activation pathways. A Random Forest classifier was trained on selected biomarkers and evaluated using leave-one-out cross-validation (LOOCV), permutation testing, and bootstrapped AUROC confidence intervals, with model interpretability assessed by SHAP values. Data are available via ProteomeXchange with identifier PXD075349.

**Results:**

Proteomic comparison identified a compact panel of proteins distinguishing flare from remission, characterized by a molecular polarization; flare states exhibited upregulation of COL18A1 and CSF1 (vascular and myeloid activation), while remission showed sustained expression of cytoskeletal scaffolding and immunoregulatory components, including FLNA, SH3BGRL3, and IGHG4. Cytokine analyses identified coordinated chemokine modules (CXCL9, CCL2, CCL3, and CCL13) preferentially upregulated during flare. The machine-learning model achieved robust internal discrimination with a mean AUROC of 0.96. Notably, a COL18A1 normalized protein expression cut-off yielded 100% specificity and 87.5% sensitivity, acting as an objective ‘rule-in’ adjunct for active disease. Normalized protein expression (NPX) cut-off yielded 100% specificity and 87.5% sensitivity, acting as an objective “rule-in” adjunct for active disease.

**Conclusions:**

This study establishes a parsimonious 5-protein biosignature of candidate leads (COL18A1, HYOU1, IGHG4, FLNA, and SH3BGRL3) that effectively captures the multifactorial pathophysiology of SLE flare. By anchoring discovery in a systematically under sampled Indian population, this work enhances global diversity in lupus biomarker research and establishes a scalable, AI-driven framework for precision assessment of disease activity.

## 1 Introduction

Systemic lupus erythematosus (SLE) is a complex autoimmune disease characterized by multisystem involvement, unpredictable flares, and variable remission states. Despite significant advances in clinical management, the molecular mechanisms underlying these disease exacerbations remain incompletely defined, which limits the development of reliable biomarkers for monitoring and predicting flares (1). While existing clinical assessment instruments, including the British Isles Lupus Assessment Group (BILAG) scoring framework, offer standardized quantification of disease activity, these tools remain largely disconnected from high-dimensional molecular platforms that could facilitate personalized therapeutic strategies. In this investigation, we performed disease activity stratification using the Easy-BILAG instrument, a streamlined, validated derivative of the original system that enables standardized evaluation across multiple organ systems (2).

To bridge this gap in SLE, our methodological approach integrates liquid chromatography-tandem mass spectrometry (LC-MS/MS)-based plasma proteomics with targeted, multiplexed cytokine quantification. This synergistic application of orthogonal platforms permits granular assessment of lineage-specific immunological mediators while simultaneously providing comprehensive surveillance of the circulating proteome. Recognizing documented inter-population variability in cytokine biomarker validity across genetic ancestries, we incorporated parallel cytokine analysis to supplement hypothesis-free proteomic screening in this South Asian study population. By anchoring discovery in an integrated AI-driven pipeline, we move beyond univariate models toward a systems-level understanding of SLE pathophysiology, elucidating its interconnected networks (3).

Critically, this work addresses a major knowledge deficit in global SLE biomarker research by focusing on a systematically underrepresented Indian population. Germline genetic variations characteristic of South Asian cohorts, including polymorphisms in major histocompatibility complex (HLA) loci, complement components, and immunoglobulin Fc receptor (FCGR) alleles, may modulate clinical endophenotypes and molecular signatures. This investigation constitutes the inaugural study to couple standardized Easy-BILAG stratification with an integrated omics architecture in an Indian SLE population. Leveraging this experimental framework, our discovery-phase protocol aims to (i) identify distinct proteomic and cytokine signatures of lupus activity, (ii) validate these findings using clinically anchored stratification, and (iii) establish a parsimonious, machine learning-validated biosignature of candidate leads as a hypothesis-generating foundation for a larger longitudinal validation to predict SLE flare.

## 2 Materials and methods

A discovery-phase investigation enrolled 16 Indian patients with systemic lupus erythematosus (SLE) recruited from tertiary-level rheumatology facility. All participants fulfilled the 2019 EULAR/ACR classification criteria to ensure a standardized disease definition (4). Disease activity was quantified using the Easy-BILAG instrument, a streamlined, validated derivative of the original British Isles Lupus Assessment Group (BILAG) scoring framework, under an academic license from the University of Leeds.

To enable uniform statistical comparisons and ensure reproducible group stratification, categorical Easy-BILAG domain scores (A–E) were converted to numeric values (A = 4, B = 3, C = 2, D = 1, E = 0). Following this conversion, patients were stratified into the Flare group (n=8; defined by a Total Numeric Score ≥10 with at least one A or B domain) or the Remission group (n=8; defined by a Total Numeric Score ≤5 with all domains scoring D or E). This standardized conversion allowed for integrated multi-omic and machine learning analysis across the cohort. Individuals with significant comorbidities, including diabetes, pregnancy, malignancy, or overlap syndromes, were excluded. The study received ethical approval from the Institutional Ethics Committee (IEC1: 28/2024) and was prospectively registered with the Clinical Trials Registry of India (CTRI/2024/07/071185). Raw and processed proteomics data are publicly available in the PRIDE repository (5), under accession number PXD075349.

### 2.1 Integrated proteomic and cytokine profiling

Peripheral blood was collected in EDTA vials and processed within one hour. Plasma was isolated via dual centrifugation at 4 °C (1,500 × g for 20 minutes, followed by 1,000 × g for 10 minutes) to remove cellular debris.

- Global Proteomics: Plasma proteins were digested using a sequencing-grade trypsin-based protocol after denaturation, dithiothreitol (DTT) reduction, and iodoacetamide (IAA) alkylation. Peptides were analyzed on a Thermo Scientific Exploris 240 Orbitrap platform coupled with nano-liquid chromatography. Raw data were processed in Proteome Discoverer against the UniProt human reference proteome, applying a 1% False Discovery Rate (FDR) and requiring ≥2 unique peptides per protein identification. Instrumentation: Experiments utilized an Easy-nLC 1000 system coupled to an Orbitrap Exploris 240 mass spectrometer.
- Sample Preparation: 1 μg of peptide sample was loaded onto a 15 cm PicoFrit column with 1.9 micron resin. Chromatography: Peptides were separated using a 110-minute total runtime with a 0–38% gradient of buffer B (80% acetonitrile, 0.1% formic acid) at a flow rate of 500 nL/min. Mass Spectrometry Settings: MS1 spectra were acquired at 60,000 resolution (m/z 380-985), with a maximum injection time of 20 ms and a 300% AGC target. DIA Parameters: MS/MS scans used a 12 Da isolation window at 30,000 resolution, 40 ms injection time, and 2,000% AGC target.
- Targeted Cytokine Profiling: Lineage-specific immune mediators were quantified using the Olink 48 panel Proximity Extension Assay (PEA) platform. A targeted panel of 48 inflammatory mediators was analyzed; however, IL-4 was explicitly excluded from all subsequent computational workflows as its concentration remained consistently below the assay’s limit of detection (LOD) across the entire Indian cohort. This resulted in a final refined dataset of 44 cytokines for the integrated bioinformatics pipeline.

### 2.2 Bioinformatics and machine learning pipeline

- Software: Proteomic data were processed using DIA-NN 2.2.0 Academia software.
- Quantification: Match-between-runs (MBR) was enabled, with peptide length filters set between 7 and 30 amino acids.
- Normalization: Median-Based Quantile Normalization (MBQN) was applied to minimize technical variability, such as ionization efficiency and batch-related signal drift, while maintaining relative abundance trends.
- Imputation: Missing values were addressed using a downshift of 1.8 and a width of 0.3.
- Data Processing: Proteomic data were processed using DIA-NN 2.2.0 Academia software. Match-between-runs (MBR) was enabled, with peptide length filters set between 7 and 30 amino acids. Median-Based Quantile Normalization (MBQN) was applied to minimize technical variability, such as ionization efficiency and batch-related signal drift. Missing values were addressed using a downshift of 1.8 and a width of 0.3. To address the high-dimensional nature of the integrated dataset (p=66 significant analytes) relative to the cohort size (n=16), we implemented an explainable AI pipeline designed to minimize overfitting while maximizing diagnostic interpretability.
- Feature Selection: To address the high-dimensional nature of the integrated dataset relative to the cohort size, we implemented an explainable AI pipeline designed to minimize overfitting. Initial statistical filtering was performed using Welch’s t-test and effect-size calculation (Cohen’s d > 0.8). Subsequently, the selected features were standardized using Z-score normalization, and Recursive Feature Elimination (RFE) was employed to identify a parsimonious 5-protein biosignature exhibiting optimal discriminative power.
- Model Validation: To mitigate the limitations of the pilot cohort size (n=16) and address potential overfitting, we implemented several rigorous validation strategies. The Random Forest classifier was evaluated using Leave-One-Out Cross-Validation (LOOCV) to maximize training data utility. To ensure the observed performance exceeded random chance, we performed 1,000-fold permutation testing, yielding a highly significant empirical p-value of 0.0030. Furthermore, 95% confidence intervals (CI) were derived for all performance metrics (Accuracy, Sensitivity, Specificity, and AUROC) using 1,000-sample bootstrap resampling to transparently estimate model stability. Furthermore, a repeated stratified 4-fold cross-validation (50 iterations) was used as a ‘stress test’ to ensure the biosignature remained robust when the training set was reduced to 75% of the total cohort.
- Interpretability: Model transparency was achieved through SHAP (SHapley Additive exPlanations) value analysis, quantifying the global and local contribution of each biomarker to the classification of flare versus remission. Additionally, optimal diagnostic thresholds for continuous biomarker levels (e.g., COL18A1) were derived by maximizing Youden’s J statistic (Sensitivity + Specificity - 1) on the Receiver Operating Characteristic (ROC) curves.
- Statistics and Computational Environment: For this discovery-centric analysis, a primary significance cutoff of p<0.05 was applied, with effect sizes (log_2_FC) and Benjamini-Hochberg False Discovery Rate (FDR) corrections reported to support robustness. All machine learning algorithms and statistical tests were implemented in Python (v3.10) using the *scikit-learn*, *scipy*, and *shap* libraries.

**Figure 1:**
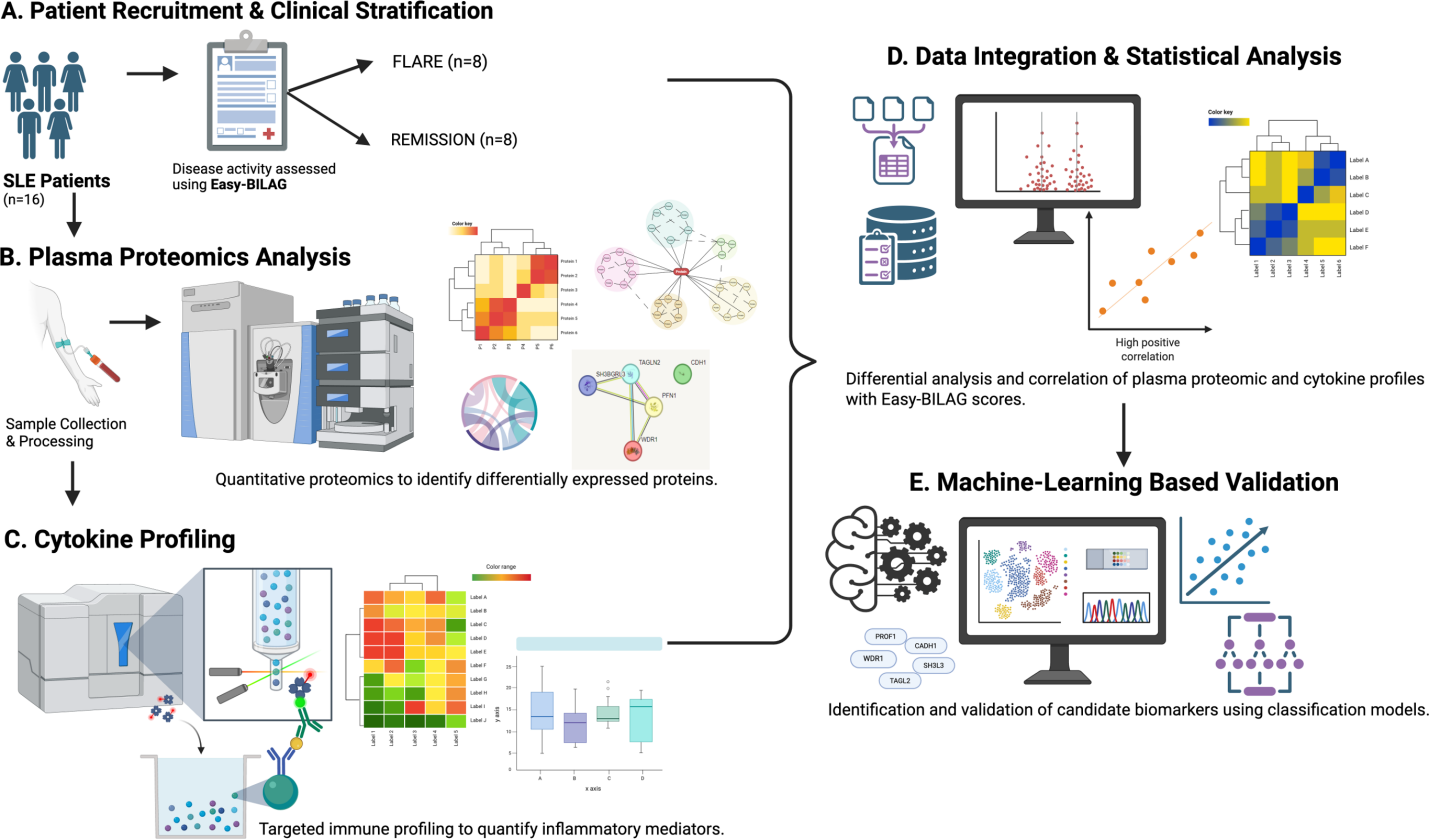
Schematic overview of the integrated multi-omics and explainable AI (XAI) discovery pipeline. This figure illustrates the study’s progression across four key phases: **(A)** Clinical Phenotyping, where theIndian SLE cohort isstratified into Flare and Remission states using Easy-BILAG indices; **(B)** High-Dimensional Proteomics, involving parallel discovery-phase LC-MS/MS and targeted Olink 48 panel Proximity Extension Assay (PEA); **(C)** Computational Analysis, focusing on data normalization, differential expression, and protein-protein interaction networking; and **(D)** Machine Learning Framework, utilizing Recursive Feature Elimination (RFE) for feature selection, followed by Random Forest classification with Leave-One-Out Cross-Validation (LOOCV) and SHAP-based interpretability. Created in BioRender. Srivastava, T. (2026) https://BioRender.com/cvyeg11.

The comprehensive integration of the experimental platforms and the hierarchical computational architecture employed in this discovery-phase study is summarized in **Figure 1**.

### 2.3 Statistical assessment of confounding variables

To ensure the identified proteomic signatures reflect intrinsic disease biology rather than therapeutic intervention, we assessed medication exposure across the cohort. Statistical evaluation confirmed that exposure to standard treatments, including Hydroxychloroquine, Mycophenolate mofetil, and Corticosteroids, was comparable between flare and remission groups. Furthermore, the lack of drug-specific clustering in unsupervised Principal Component Analysis (PCA) suggests that the observed molecular polarization is driven by disease state rather than differential treatment.

## 3 Results

### 3.1 Clinical and demographic characterization

The discovery cohort consisted of 16 Indian SLE patients, distributed evenly into active flare (n=8) and remission (n=8) groups based on comprehensive clinical evaluation (**Table 1**). Both cohorts were demographically matched, demonstrating similar age distributions (mean 26.38 ± 4.77 vs. 25.25 ± 4.83 years) and identical gender ratios.

**Table 1:** Patient demographics.

Characteristics	Flare (n = 8)	Remission (n = 8)
Age	26.0 ± 4.28	25.25 ± 4.83
Sex (F/M)	7/1	8/0
Disease Duration	7.0 [3.5 – 9.0]	8.0 [1.0 – 5.5]
BILAG Score (Total score) median (IQR)	14.5 (IQR 12.5 – 15.5)	4.5 (IQR 4-5)
Clinical involvement		
Renal (proteinuria > 0.5 g/day)	4/8	4/8
Neuropsychiatric manifestations	2/8	2/8
Arthritis	3/8	4/8
Cutaneous lesions	1/8	2/8
Routine labs		
Haemoglobin (g/dL)	11.35 ± 1.09	10.43 ± 1.03
WBC count (109/L)	6.47 ± 2.29	6.56 ± 2.52
Platelets (109/L)	221.9 ± 100.02	187.6 ± 91.8
ESR (mm/hr)	42.5 ± 2.4	24.5 ± 13.2
CRP (mg/L)	7.13 ± 11.15	2.27 ± 1.49
Serum creatinine (mg/dL)	0.57 ± 0.13	1.22 ± 0.72
Immunology		
ANA positive	8/8	8/8
Anti-Sm	4/8	0/8
Anti-U1RNP	6/8	0/8
Complement C3 (mg/dL)	48 ± 10.1	95 ± 15
Complement C4 (mg/dL)	10 ± 3.0	25 ± 5
Medications		
Hydroxychloroquine	8/8	8/8
Mycophenolate mofetil	5/8	6/8
Cyclophosphamide	0/8	0/8
Azathioprine	1/8	1/8
Wysolone	3/8	3/8
Omnacortil	2/8	2/8
Corticosteroids	2/8	2/8
Rituximab	1/8	1/8

**Table 1** displays the study cohort’s demographic, clinical, laboratory, immunological, and treatment data by disease activity. We recruited 16 SLE patients, eight with active disease and eight in Easy-BILAG-defined clinical remission. Both groups had identical ages and sex distributions, but disease activity, laboratory data, immunological characteristics, and drug exposure differed. Patients without anti-dsDNA antibodies were excluded from quantitative analysis. Medical examination and clinical factors determined flare and remission.

To contextualize systemic proteomic alterations within the patients’ physical presentations, disease activity was quantified and organ-stratified using the Easy-BILAG scoring system (**Table 2**). The flare group exhibited significantly higher cumulative disease activity (Total Score ≥10) compared to the remission cohort (Total Score ≤5). Specifically, active flare presentations were predominantly driven by musculoskeletal and mucocutaneous involvement, with a distinct subgroup exhibiting renal and hematological manifestations. Conversely, the remission cohort was defined by ‘D’ or ‘E’ scores across all organ systems, indicating minimal or absent activity; minor numerical variations in their total scores were solely due to these inactive domains and did not reflect clinically active disease.

**Table 2:** Easy BILAG score: FLARE (1F-8F) and remission (9R-16R).

Patient group	General	Mucocutaneous	MSK	Renal	Haematologic al	Neuropsychiatric	Opthalmic	GI	Cardio respiratory	Total BILAG score
1F	B-3	B-3	A-4	C-2	E-0	E-0	E-0	D-1	E-0	13
2F	E-0	C -2	A-4	A-4	D-1	E-0	E-0	D-1	B-3	15
3F	E-0	E-0	E-0	A-4	B-3	B-3	E-0	E-0	E-0	10
4F	B-3	A-4	B-3	E-0	D-2	E-0	E-0	E-0	E-0	12
5F	E-0	A-4	A-4	D-1	C-2	E-0	E-0	E-0	A-4	15
6F	E-0	B-3	E-0	A-4	C-3	E-0	E-0	E-0	A-4	14
7F	D-1	B-3	A-4	A-4	C-2	E-0	A-4	E-0	E-0	18
8F	E-0	A-4	D-1	A-4	B-3	A-4	E-0	E-0	E-0	16
1R	E-0	E-0	C-2	D-1	C-2	E-0	E-0	E-0	E-0	5
2R	E-0	D-1	D-1	E-0	D-1	D-1	E-0	E-0	E-0	4
3R	E-0	D-1	D-1	D-1	D-1	D-1	E-0	E-0	E-0	5
4R	E-0	D-1	D-1	E-0	C-2	E-0	E-0	E-0	E-0	4
5R	E-0	D-1	D-1	D-1	D-1	E-0	E-0	E-0	D-1	5
6R	D-1	D-1	D-1	D-1	E-0	E-0	E-0	E-0	E-0	4
7R	E-0	E-0	E-0	D-1	D-1	E-0	E-0	E-0	E-0	2
8R	E-0	D-1	D-1	D-1	D-1	E-0	E-0	E-0	D-1	5

**Table 2** depicts the EASY-BILAG Score organ-based stratification based on actual score and below-score (A–E), which were converted into numeric values (A = 4, B = 3, C = 2, D = 1, E = 0) to ensure uniformity. Grouping into flare and remission was based on these standardized scores.

This detailed clinical mapping provided a crucial framework to understand how global molecular signatures align with localized manifestations, which was subsequently evaluated via domain-wise and stratified correlation analyses (**Supplementary Figures 2**–**4**). Importantly, medication exposure, including hydroxychloroquine, mycophenolate mofetil, and corticosteroids, wasstatistically comparable between thetwo groups. Theabsenceof consistent drug-related clustering in subsequent analyses suggests that the identified molecular signatures were driven by intrinsic disease biology rather than differential treatment exposure.

### 3.2 Integrated proteomic landscape and molecular polarization

Comparative plasma proteomic analysis via LC-MS/MS identifies a distinct molecular polarization between flare and remission states (**Supplementary Figure 1**). Statistical evaluation reveals 22 proteins with significant alterations (p < 0.05), comprising 6 upregulated and 16 downregulated markers in the flare cohort (**Table 3**).

**Table 3:** Significantly differentially expressed proteins between flare and remission groups.

Proteins	Gene names	Log2 fold change	*pValue*	Direction (w.r.t flare)
WDR1_HUMAN	WDR1	-1.94237	0.029128	Downregulated
HPTR_HUMAN	HPR	1.035462	0.048187	Upregulated
ANGT_HUMAN	AGT	0.703626	0.03014	Upregulated
IGHG4_HUMAN	IGHG4	-2.78059	0.000959	Downregulated
AMBP_HUMAN	AMBP	0.553323	0.03503	Upregulated
G3P_HUMAN	GAPDH	-0.89158	0.035212	Downregulated
ENOA_HUMAN	ENO1	-0.89576	0.036945	Downregulated
PROF1_HUMAN	PFN1	-1.35233	0.044663	Downregulated
HSP7C_HUMAN	HSPA8	-1.78067	0.043359	Downregulated
CADH1_HUMAN	CDH1	1.193486	0.022059	Upregulated
KPYM_HUMAN	PKM	-0.97564	0.016884	Downregulated
FLNA_HUMAN	FLNA	-2.13504	0.005691	Downregulated
MOES_HUMAN	MSN	-0.72961	0.041811	Downregulated
PDIA3_HUMAN	PDIA3	-0.71613	0.034033	Downregulated
COR1A_HUMAN	CORO1A	-1.7786	0.045495	Downregulated
TAGL2_HUMAN	TAGLN2	-2.51505	0.042245	Downregulated
COIA1_HUMAN	COL18A1	1.090499	0.001429	Upregulated
1433Z_HUMAN	YWHAZ	-1.54078	0.025585	Downregulated
MA2A1_HUMAN	MAN2A1	0.845924	0.017566	Upregulated
URP2_HUMAN	FERMT3	-2.32551	0.019934	Downregulated
SH3L3_HUMAN	SH3BGRL3	-2.18789	0.004659	Downregulated
HYOU1_HUMAN	HYOU1	0.979026	0.004768	Upregulated

**Figure 2:**
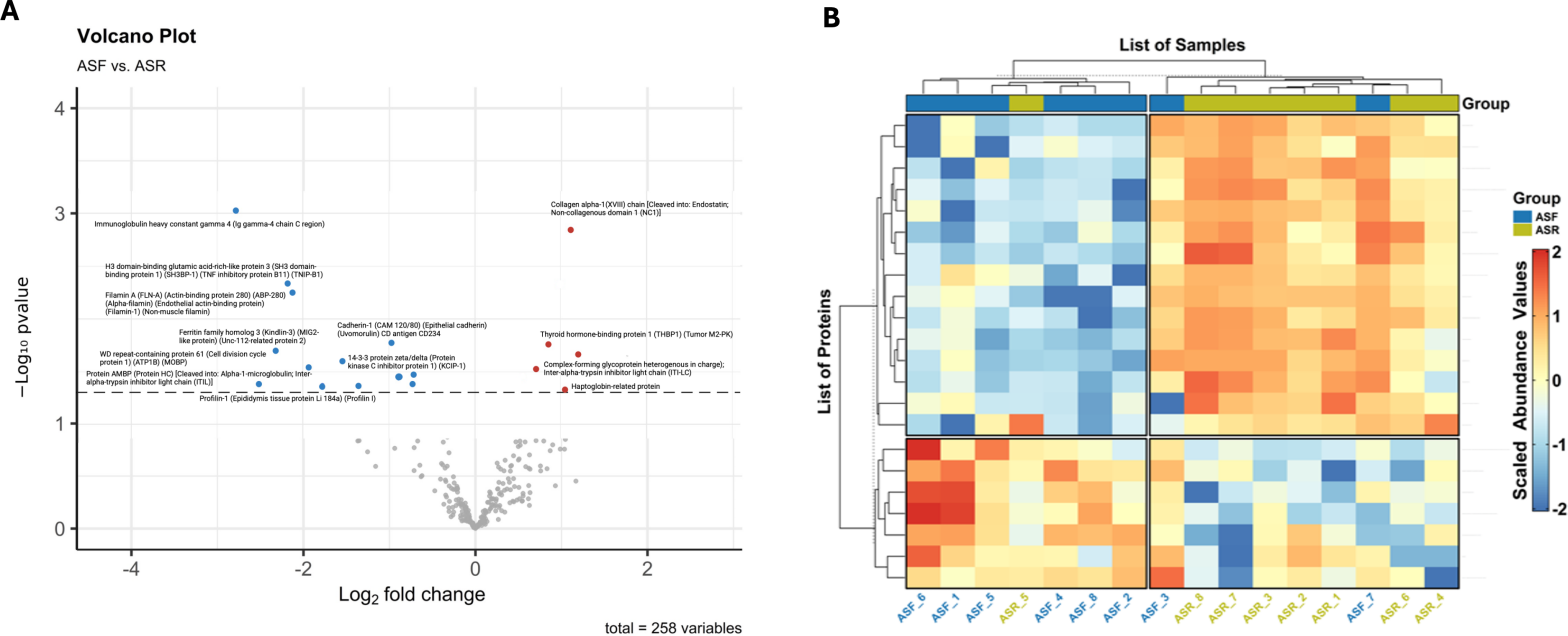
Differential plasma proteomic signatures distinguish flare and remission states in SLE. **(A)** Volcano plot depicting the initial, unannotated differential expression landscape across all quantified plasma proteins in lupus flare (ASF) vs. remission (ASR), with log_2_ fold change on the x-axis and –log_10_ p-value on the y-axis. Significant proteins (p < 0.05, red) include upregulated COL18A1 (+1.09, p = 0.0014) and HPR, and downregulated IGHG4 (– 2.78, p = 0.0009), FLNA, TAGLN2, FERMT3, SH3BGRL3 (gray: non-significant). **(B)** Heatmap shows Z-score normalized abundances of significant proteins across samples (flare: blue, remission: yellow-green). Colors indicate levels (blue low, red high); hierarchical clustering separates groups and reveals flare-associated elevated clusters.

The active flare state is characterized by vascular and acute-phase upregulation, specifically marked by increases in COL18A1 (log_2_FC = 1.09, p = 0.0014) and HPR (**Figure 2A**).

Conversely, clinical remission is associated with the preservation of structural and immunoregulatory components, including IGHG4 (log_2_FC = –2.78, p = 0.0009), FLNA, and SH3BGRL3, which are suppressed during active disease.

Unsupervised hierarchical clustering of these differentially expressed proteins effectively separated the flare (ASF) and remission (ASR) samples into distinct molecular endotypes (**Figure 2B**), aligning closely with known lupus pathways of vascular dysfunction and myeloid activation and suggesting potential ancestry-independent relevance in this Indian cohort.

In flare states, COL18A1 was upregulated (log_2_ fold change = 1.09, p = 0.0014), while IGHG4 was markedly downregulated (–2.78, p = 0.0009), indicating clear abundance differences. Assessed at p<0.05 without correction, these exploratory findings require validation in larger cohorts.

Mass spectrometry revealed p<0.05 differences in protein expression between lupus flare/remission; the data included UniProt accessions, gene symbols, descriptions, log-FC (positive for flare upregulation, negative for remission downregulation), and p-values. For exploratory analysis, 22 proteins met the threshold, and Benjamini-Hochberg FDR prioritized COL18A1, CSF1 as strongly associated with disease activity.

**Table 3** shows that 22 proteins were significantly differentially expressed between the flare and remission groups (p < 0.05). Of these, 7 were upregulated in flare; however, 15 were downregulated. Notably, IGHG4, FLNA, TAGLN2, and FERMT3 showed >2-fold downregulation in flare, whereas COL18A1 and HPR were upregulated.

Differentially expressed proteins clustered into three major functional categories:

- Structural/cytoskeletal proteins (e.g., FLNA, TAGLN2, FERMT3) were consistently downregulated during flare, suggesting impaired cellular integrity.
- Immune-regulatory proteins (e.g., IGHG4, SH3BGRL3) showed suppression, reflecting dampened regulatory circuits.
- Endothelial and stress-response proteins (e.g., COL18A1, HPR) were upregulated, indicating vascular activation and acute-phase responses.

**Figure 3:**
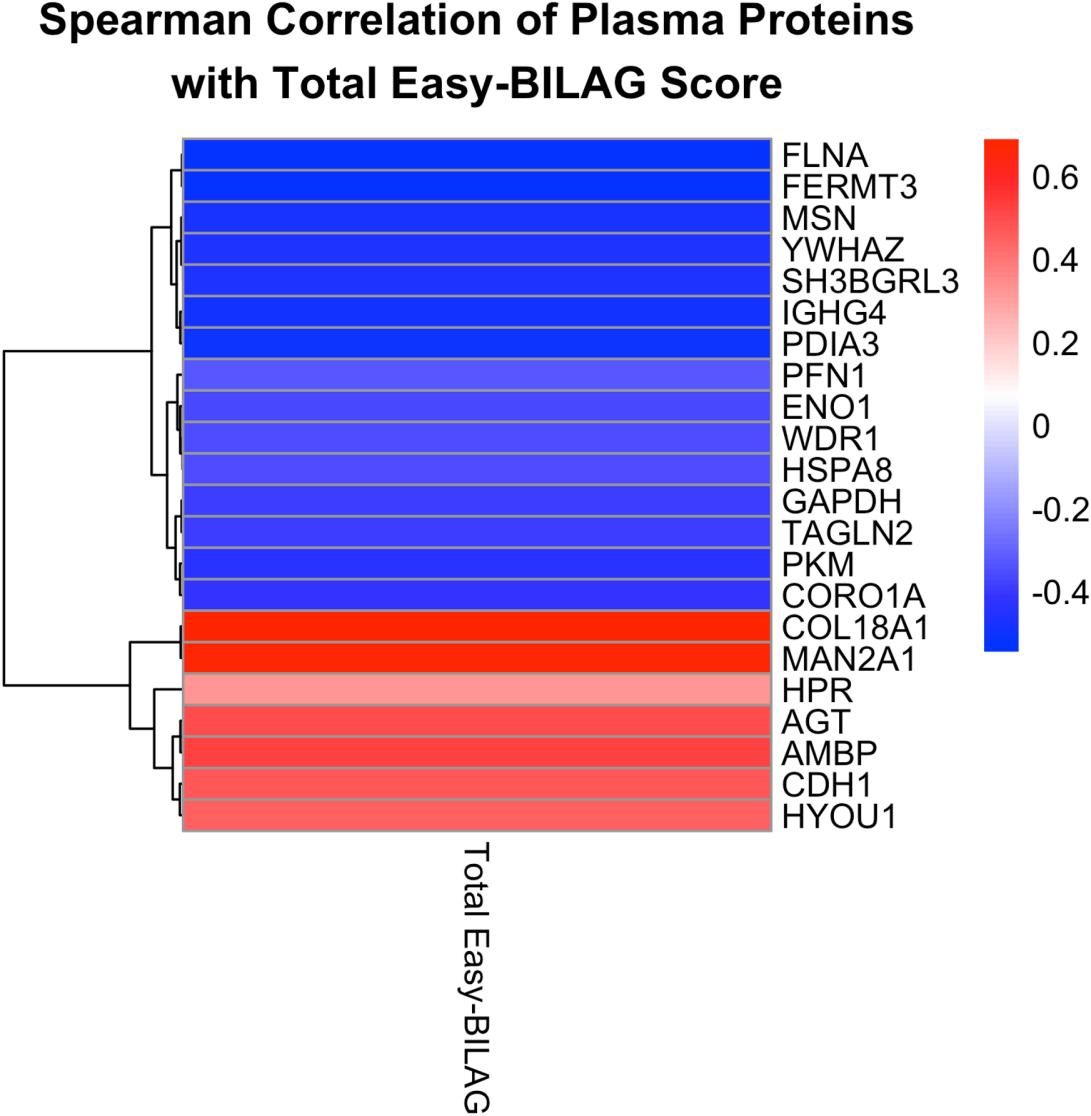
Spearman correlation matrix of the SLE flare-associated proteome and clinical disease activity.

**Figure 4:**
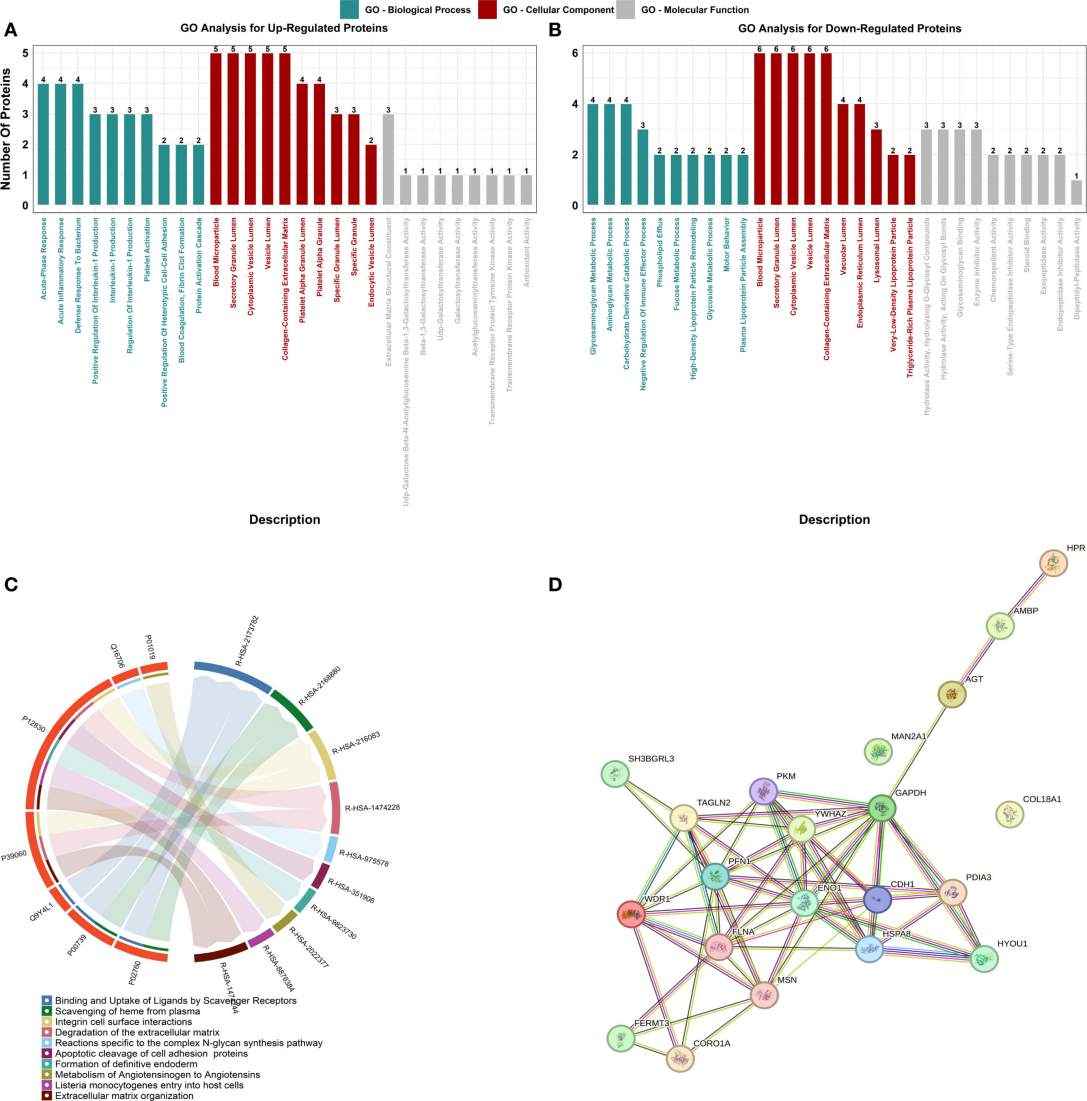
Systems-level protein-protein interaction (PPI) network and functional biological clustering in lupus flare. **(A)** Gene Ontology classified enriched terms for flare-upregulated proteins: Biological Process (teal) - acute-phase/pro-inflammatory; Cellular Component (dark red) - vesicular lumens/platelet granules; Molecular Function (gray). Pathways highlighted inflammation, platelet activation, integrin adhesion, and vascular dysfunction. Downregulated categories included enriched lipid remodeling, glycosaminoglycan synthesis, and immune regulation, thereby indicating suppressed homeostasis. **(B)** Downregulated proteins showed enriched GO terms in glycosaminoglycan biosynthesis and immunoregulation, with localizations in lipoprotein complexes and secretory granules, reflecting reduced lipid metabolism and immune responses. Enrichments: Upregulated in acute-phase (adjusted p=0.004, FDR = 0.01) and IL-1 cascades (adj p=0.007, FDR = 0.02); downregulated in glycosaminoglycan metabolism (adj p=0.009, FDR = 0.02) and lipoprotein remodeling (adj p=0.012, FDR = 0.03). **(C)** Reactome pathways for flare emphasized ECM organization, leukocyte adhesion via integrins, and scavenger activity, denoting vascular changes, immune activation, and oxidative stress. Downregulated pathways focused on lipid metabolism and immune circuits, aligning with homeostatic suppression. **(D)** PPI network depicting interactions among differentially expressed proteins, with nodes representing genes and edges representing associations. Clusters include cytoskeletal (FLNA, FERMT3, TAGLN2, MSN), metabolic (GAPDH, PKM, ENO1), and immune (HSPA8, PDIA3, YWHAZ, COL18A1), illustrating coordinated shifts in lupus activity. Unsupervised PCA on all quantified plasma proteins evaluated the global variance structure. Biplot separates flare (ASF, blue circles) and remission (ASR, yellow triangles) samples via log_2_ abundances, with Dim1 (56.7%) and Dim2 (10.1%) variance highlighting distinct signatures. Outliers suggest transitional states or limitations in the EASY-BILAG scoring system.

Directionality of differential abundance was defined by the sign of log2 fold change, with positive values indicating higher abundance in flare state, and negative values indicating lower abundance. COL18A1, linked to endothelial stress and angiogenesis (6; 7), and CSF1, a macrophage growth factor (8), align with known lupuspathwaysof vascular dysfunction and myeloid activation (9). Their prominence in this Indian cohort suggests potential ancestry-independent relevance, though validation is required. The clinical relevance of these markers and their systems-level biological organization are visually summarized in the correlation matrix (**Figure 3**) and the protein-protein interaction network (**Figure 4**), respectively.

**Figure 5:**
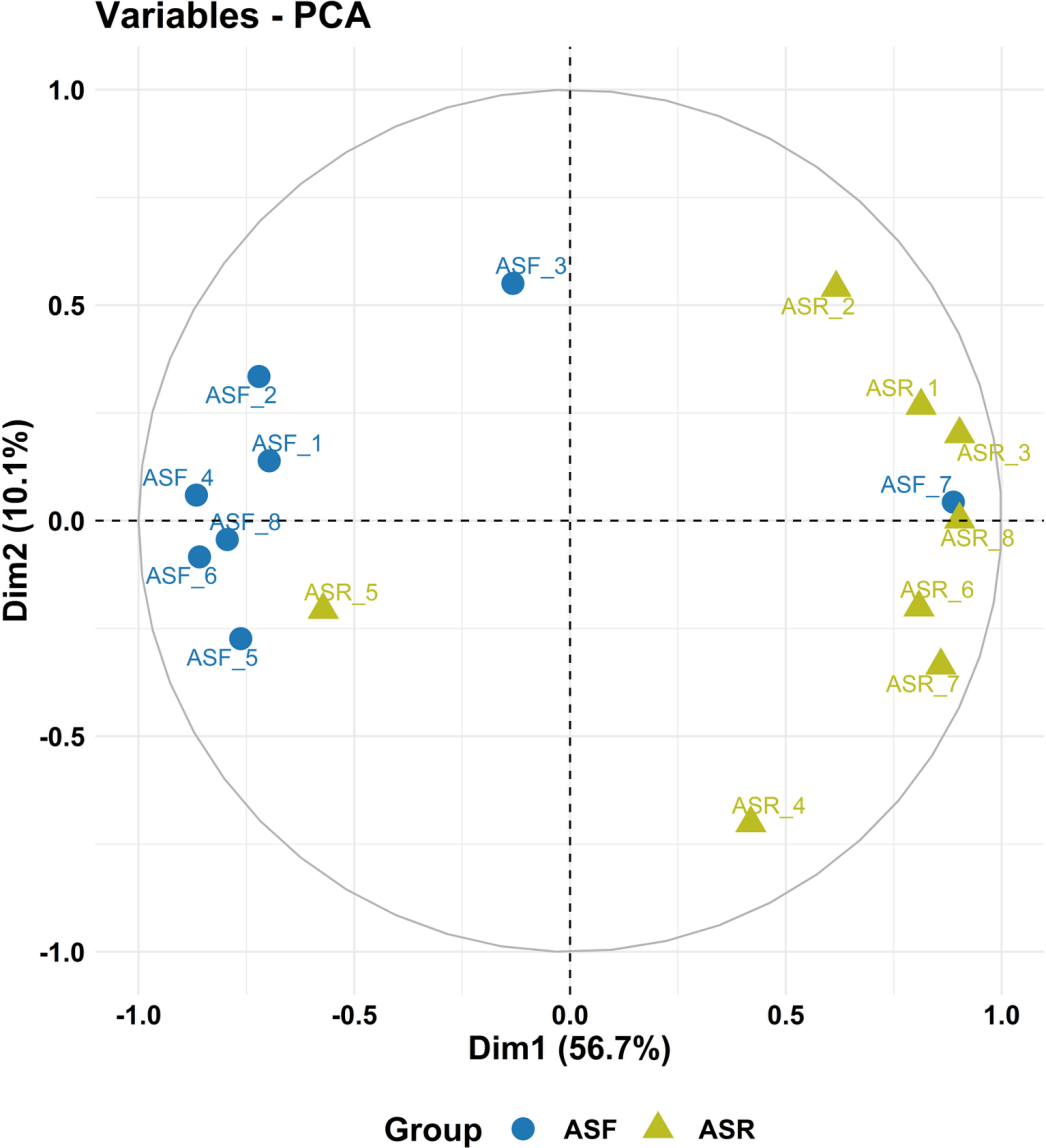
Principal Component Analysis (PCA) of proteomic profiles in flare and remission samples. After Benjamini-Hochberg (FDR<0.1), COL18A1, HPR, and IGHG4 were significant as biomarkers. COL18A1 (r=0.52, 95% CI 0.18–0.74); IGHG4 (r=-0.38, 95% CI –0.65 to –0.05), confirming cohort reliability. Biologically, COL18A1 is tied to endothelial activation in flares, and an inverse association with IGHG4 suggests immunoregulatory disruption, thereby boosting clinical relevance.

To assess the global variance and the degree of separation between clinical states, we performed Principal Component Analysis on the normalized expression data. The resulting PCA plot (**Figure 5**) demonstrates clear group-wise segregation, indicating that the integrated proteome provides a robust basis for distinguishing flare from remission.

### 3.3 Functional enrichment and systemic cytokine profiling

The heatmap (**Figure 3**) shows Spearman correlations between plasma proteins and lupus activity, according to the Total Easy-BILAG Score; blue for negative, red for positive; intensity denotes strength. Clustering highlights modules tied to flare severity; positive correlates were: COL18A1, HPR, AGT, CDH1; negative correlates were: FLNA, FERMT3, IGHG4, TAGLN2.

A pilot study revealed distinct inflammatory protein and cytokine profiles between SLE flare and remission using Olink Target 48 panel on 16 patients stratified by BILAG. Post-QC, imputation, normalization, and statistical/machine learning analyses identified discriminative biomarkers. Enrichment analyses applied p<0.05 without correction, reflecting an exploratory discovery phase; findings are hypothesis-generating and need larger cohort validation.

To complement the global proteomic findings, targeted immune profiling of 44 inflammatory mediators was performed using the Olink Proximity Extension Assay (PEA) (raw values in **Supplementary Table 2**). This integrated approach identified a coordinated module of cytokines and chemokines that were significantly elevated in the flare cohort, reflecting a heightened systemic inflammatory tone.

- Myeloid Activation and Recruitment: Flare states were characterized by significant elevations in CSF1 (M-CSF), CCL13 (MCP-4), and CXCL9, signaling robust myeloid lineage activation and chemotactic signaling.
- Interferon-Driven Signaling: The presence of elevated CXCL9 further underscores the activation of the interferon-gamma (IFN-γ) axis, which is a hallmark of systemic lupus erythematosus pathogenesis.
- Exclusion of Non-Informative Analytes: IL-4 was excluded from the final analysis because concentrations remained consistently below the assay’s limit of detection (LOD) across the Indian cohort, thereby restricting the computational workflow to high-confidence analytes.

**Figure 6:**
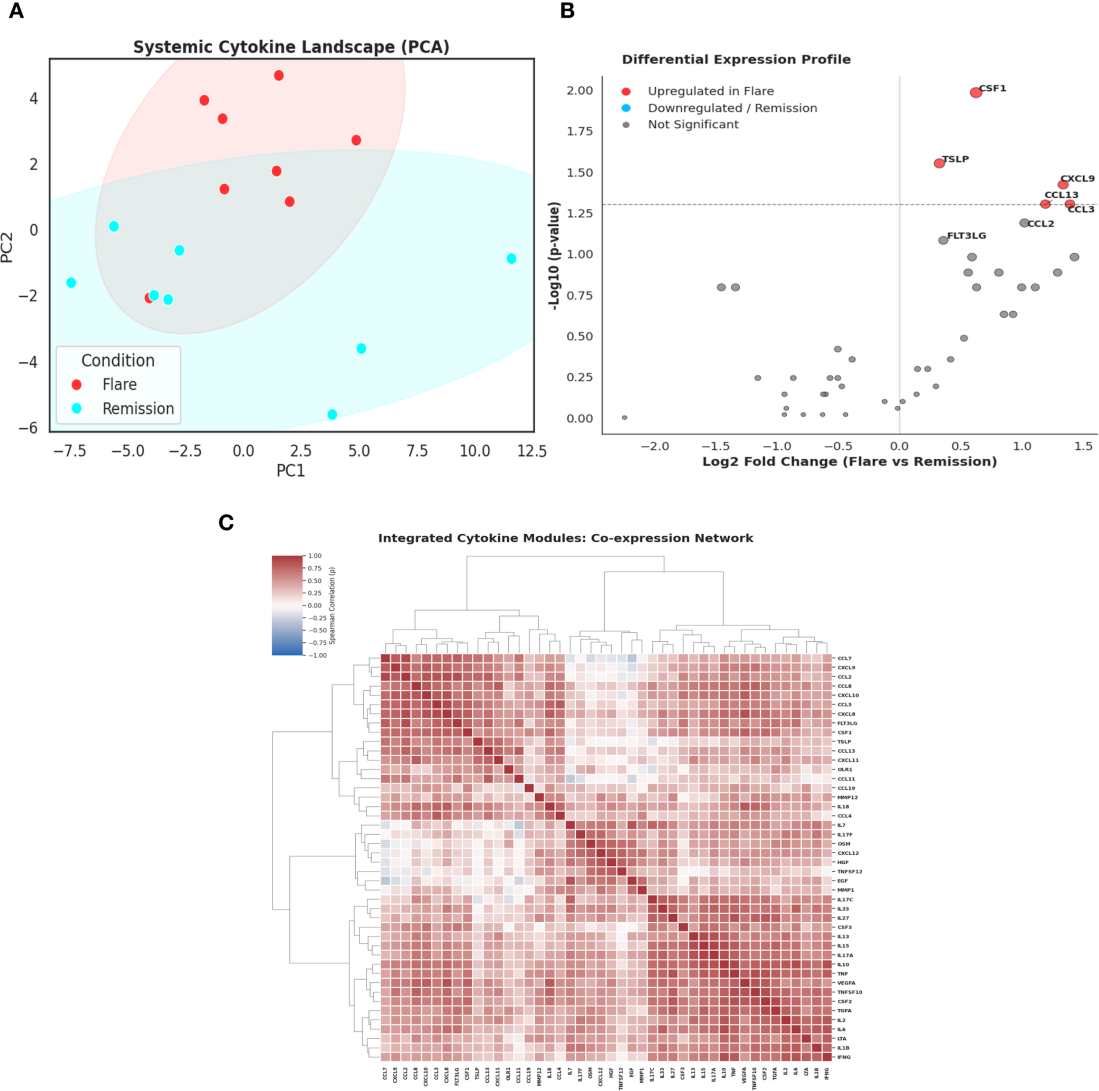
Cytokine profile. **(A)** Systemic Cytokine Landscape (PCA): PCA illustrates partial separation of cytokine profiles in 16 SLE patients between flare (red) and remission (cyan) states along PC1 and PC2. Points denote patients, with ellipses highlighting group distributions, revealing a flare-induced shift despite some overlap. **(B)** Differential Expression Profile of Cytokines: Volcano plot plots log2 fold change (flare vs. remission) against –log10 p-value; red-highlighted cytokines (CSF1, TSLP, CXCL9, CCL2, CCL3, CCL13) exhibit significant upregulation in flares. **(C)** Integrated Cytokine Modules: Heatmap of hierarchical-clustering Spearman correlations among cytokines shows positive (warm) and negative (cool) associations, identifying modules such as CCL2/CCL3/CCL13, CXCL9, CSF1, and FLT3LG, which form inflammatory networks tied to SLE activity.

The overall architecture of the systemic cytokine environment, the identified co-expression modules, and the statistical distribution of these inflammatory mediators across the clinical groups are visualized in **Figure 6**, confirming a robust molecular transition associated with the flare stat.

In the global cytokine architecture, PCA of the cytokine matrix revealed partial separation between flare and remission along the first two components, with most flare samples clustering in the upper-right region and remission samples dispersed toward the opposite quadrant (**Figure 6A**: “Systemic Cytokine Landscape”); although ellipses overlapped, the centroid shift suggests coordinated cytokine reprogramming during flares via composite chemokine-cytokine modules rather than isolated single-marker changes, consistent with prior reports on SLE flares.

The co-expression network structure, shown in the hierarchical correlation heatmap, displayed dense positive co-expressions among most cytokines, with submodules enriched for CCR2/CCR5-binding chemokines(CCL2, CCL3, CCL13), interferon-linked chemokines(e.g., CXCL9, CXCL10), and growth/angiogenic factors (e.g., FLT3LG, CSF1, VEGF-family members) (**Figure 6B**: “Differential Expression Profile”), aligning with prior work implying CCR2-ligands, and CXCR3-chemokines(CXCL9/10) as quantitative correlates of SLE activity and renal involvement (10). (**Figure 6C**: “Integrated Cytokine Modules”), mirroring previously described interferon-regulated and myeloid-activation chemokine clusters in SLE (11) and supporting flare biology as a coordinated pathway activation across chemotaxis, myelopoiesis, and tissue remodeling. Differentially expressed cytokines, identified in the volcano plot, formed a limited but biologically coherent set upregulated in flare versus remission; with CSF1, TSLP, CXCL9, and chemokines CCL2/CCL3/CCL13 exhibiting the largest positive log2 fold changes and lowest p-values.

Although initial differential expression analyses were reported at p<0.05, we applied Benjamini-Hochberg false discovery rate (FDR) correction to account for multiple testing. After FDR adjustment (q<0.1), COL18A1, HPR, and IGHG4 remained significant, underscoring their robustness as candidate biomarkers. Other proteins and cytokines are reported as hypothesis-generating signals requiring validation.

**Figure 7:**
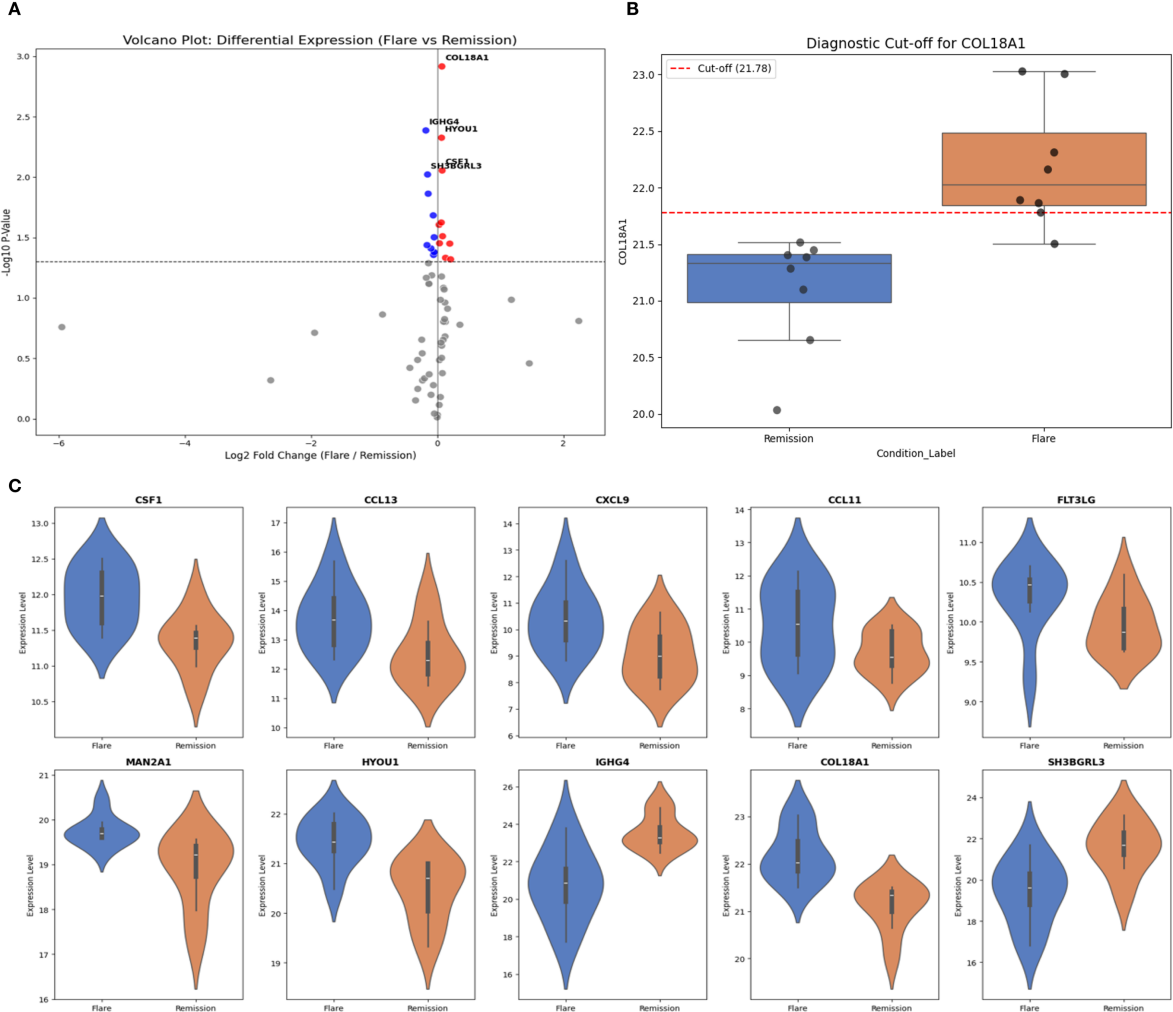
Correlation-based mechanism networks. **(A)** A secondary volcano plot was generated specifically from the refined dataset utilized for ML validation. This visualizes the statistical separability of the optimized markers, highlighting the dominance of COL18A1 (upregulated, red) and IGHG4 (downregulated, blue) as key drivers of the AI classification. **(B)** Box plot contrasts normalized COL18A1 abundances between remission (blue, n=8) and flare (orange, n=8) groups. Youden’s Index-derived cut-off (>21.78 Log2, red dashed line) yields 100% specificity (no false positives) and 87.5% sensitivity (7/8 true positives), enabling reliable flare detection. **(C)** Violin plots illustrate biomarker levels in flare (orange) vs. remission (blue), demonstrating marked differential expression indicative of flare-specific markers.

Building on the robust systemic separation observed in the principal component space (**Figure 5**), we isolated the highest-performing individual features to evaluate their standalone clinical utility. Notably, COL18A1 emerged as a dominant marker of vascular stress, prompting us to assess its absolute discriminative potential at an individual patient level (**Figure 7**).

### 3.4 Isolation and clinical correlation of candidate biomarkers

Recognizing the clinical heterogeneity of the cohort, we performed organ-specific correlation analyses using Easy-BILAG domains. These analyses demonstrated that candidate markers track with specific manifestations; for example, COL18A1 expression mirrored the severity of renal involvement, acting as a molecular surrogate for organ-specific flare intensity.

**Figure 8:**
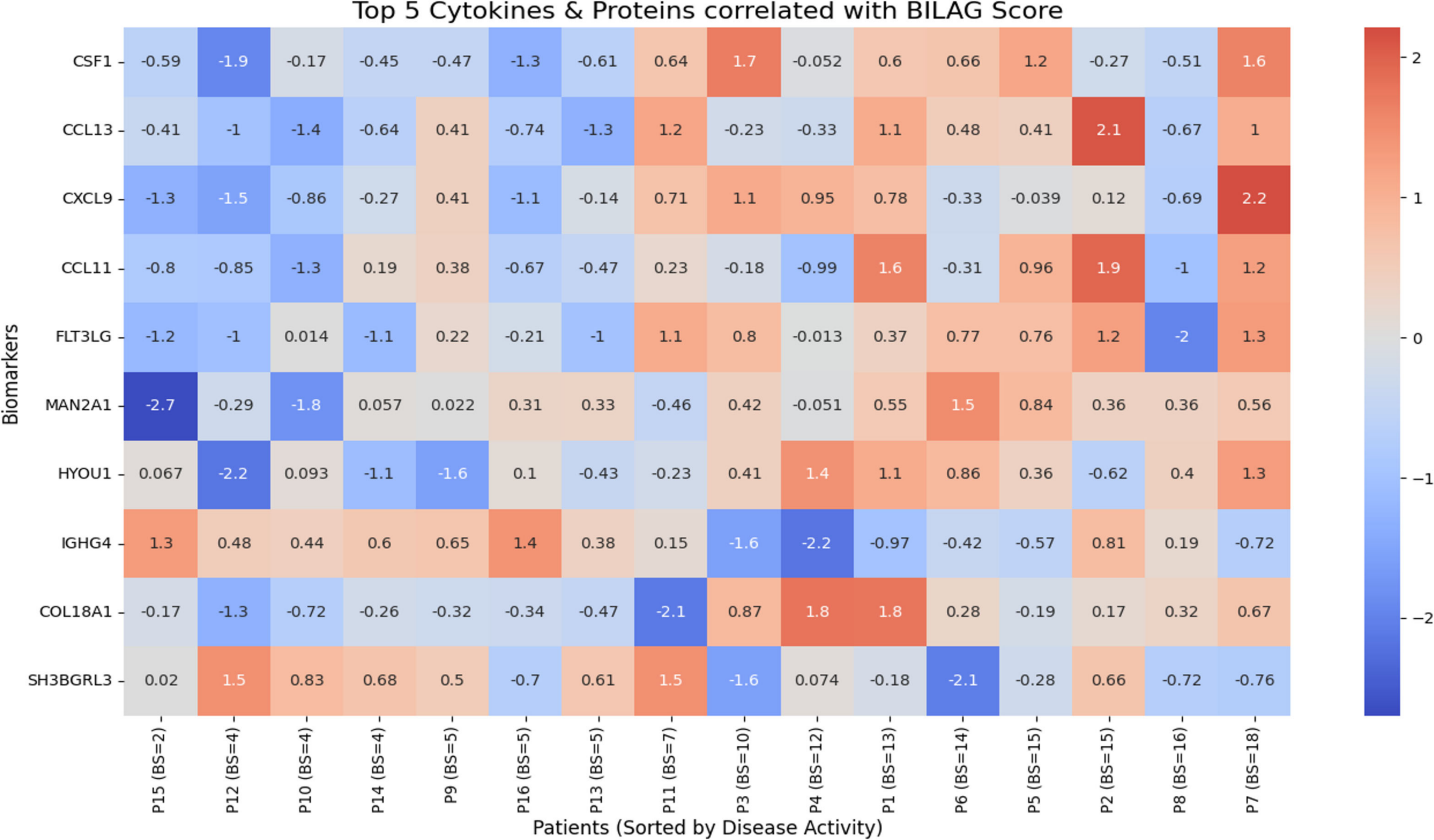
Clinical correlation. Heatmap depicts top 5 biomarkers correlated with Easy-BILAG score. Pearson analysis reveals positive links for HYOU1 (r=0.62, p=0.011) and CSF1 (r=0.61, p=0.012), negative for IGHG4 (r=-0.56) and SH3BGRL3 (r=-0.52), endorsing non-invasive activity proxy.

Machine learning analyses were conducted as discovery-phase exploratory models to assess the discriminatory potential of proteomic and cytokine features. To determine the clinical utility of the vascular-endothelial signature, we performed ROC analysis on the lead marker, COL18A1. The resulting curve and the identification of a 100% specific ‘rule-in’ threshold of 21.78 (Log2) are presented in **Figure 7**, providing a molecular basis for objective flare confirmation. Beyond its diagnostic accuracy, COL18A1 expression levels demonstrated a significant quantitative relationship with clinical disease activity. As shown in **Figure 8**, the marker’s elevation mirrored the severity of organ-specific involvement, particularly within the renal domain, reinforcing its role as a molecular surrogate for SLE flare intensity.

While these markers effectively distinguished binary flare states, we further examined whether their circulating abundance tracked quantitatively with overall disease severity. Correlation analysis confirmed that our top candidate biomarkers function as dynamic biological surrogates, mirroring the continuous Total Easy-BILAG scores (**Figure 8**).

### 3.5 Machine learning validation and diagnostic interpretability

**Figure 9:**
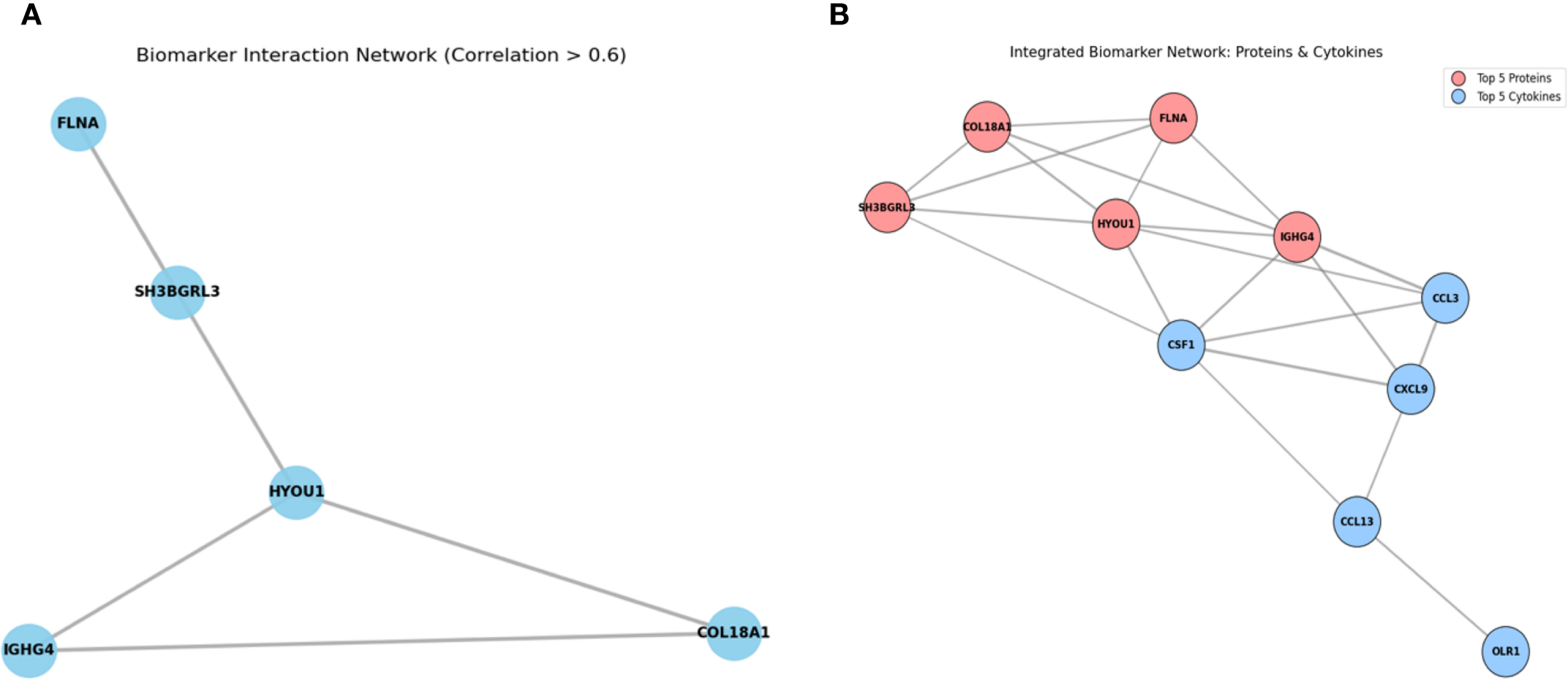
Correlation-based mechanism networks. **(A)** Network graph depicts biomarkers correlated >0.6, centered on HYOU1, FLNA, SH3BGRL3, IGHG4, COL18A1; linear structure implies core pathway, revealing SLE mechanisms for targeted therapies. **(B)** Integrated Biomarker Network links top proteins (pink) and cytokines (blue) via nodes such as CSF1, COL18A1, and IGHG4; the interconnected structure highlights SLE complexity, with HYOU1 as a key regulator for network-targeted therapies.

**Figure 10:**
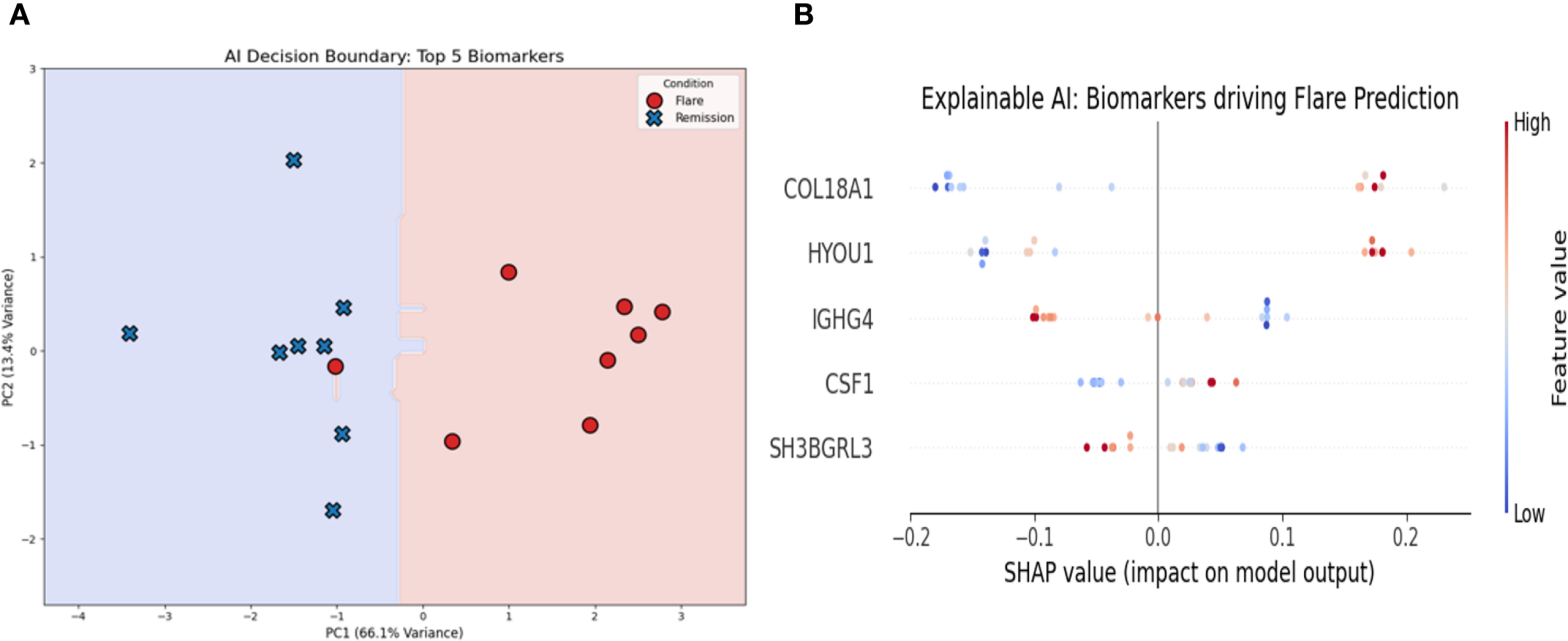
Modeling/Interpretability. **(A)** The PCA plot uses top 5 biomarkers to separate SLE flare (red circles) and remission (blue crosses), with PC1 (66.1%) and PC2 (13.4%) accounting for the variance; AI boundaries divide groups. One flare outlier suggests biological heterogeneity or proteome lag; it requires longitudinal validation, yet biomarkers remain discriminatory. **(B)** SHAP plot reveals feature influences on flare prediction, with elevated COL18A1 exhibiting a strong positive influence, increasing AI reliability for SLE.

To establish a high-performance diagnostic framework for SLE flare prediction, we implemented an AI-driven classification pipeline utilizing the integrated proteomic and cytokine features. To explore the biological interconnectedness of our lead markers and their integration with systemic cytokine signatures, we constructed correlation-based mechanism networks (**Figure 9**), revealing a tightly linked inflammatory-vascular axis. The spatial segregation of clinical samples and the individual contribution of each biomarker to the model’s diagnostic logic are visualized using PCA and SHAP interpretability analysis (**Figure 10**; see also **Supplementary Figure 6** for detailed AI decision boundaries). To evaluate whether the model’s performance was an artifact of the LOOCV methodology, a secondary sensitivity analysis was conducted as a ‘stress test.’ This involved repeated stratified 4-fold cross-validation (50 iterations), which reduced the training set size to 75% per fold (n=12). The model maintained a robust mean accuracy of 91.75% and a mean AUROC of 0.97, confirming that the identified proteomic signature provides a generalizable and stable signal even under stricter validation conditions.

- Feature Selection and Dimensionality Reduction: Using Recursive Feature Elimination (RFE), we narrowed the initial pool of 66 significant analytes down to a parsimonious 5-protein biosignature comprising COL18A1, HYOU1, IGHG4, FLNA, and SH3BGRL3. This lean model was designed to maximize diagnostic accuracy while minimizing the risk of overfitting inherent in small-cohort clinical studies.
- Classifier Performance: A Random Forest (RF) ensemble model, validated through Leave-One-Out Cross-Validation (LOOCV), achieved high accuracy of 93.75% and a mean AUROC of 0.96 (95% CI: 0.84-1.00) (**Table 4**).
- Statistical Stress-Testing: To rule out random chance, we performed 1,000-fold permutation testing, yielding a highly significant empirical p-value of 0.0030.
- Model Interpretability (SHAP): Local and global feature importance was quantified using SHAP values (**Figure 10**). COL18A1 was identified as the primary driver of flare prediction, directly linking endothelial stress to the model’s high specificity. Notably, a derived abundance threshold for COL18A1 (>21.78 Log2) demonstrated 100% specificity for “ruling in” a flare state within the Indian cohort.

**Table 4:** Performance metrics of the random forest classifier (LOOCV).

Performance metric	Value	95% CIa
Accuracy	93.75%	81.3% – 100%
Sensitivity (Recall)	87.50%	64.6% – 100%
Specificity	100.00%	100% – 100%
Precision (PPV)	100.00%	—
F1-Score	0.93	—
AUROC	0.96	0.84 – 1.00
Cohen’s Kappa (κ)	0.875	—

CI, Confidence interval; LOOCV, Leave-One-Out Cross-Validation; PPV, Positive Predictive Value; AUROC, Area Under the Receiver Operating Characteristic Curve.

**a**95% Confidence Intervals were derived using 1,000-fold bootstrap resamples to assess model stability given the pilot sample size.

**Figure 11:**
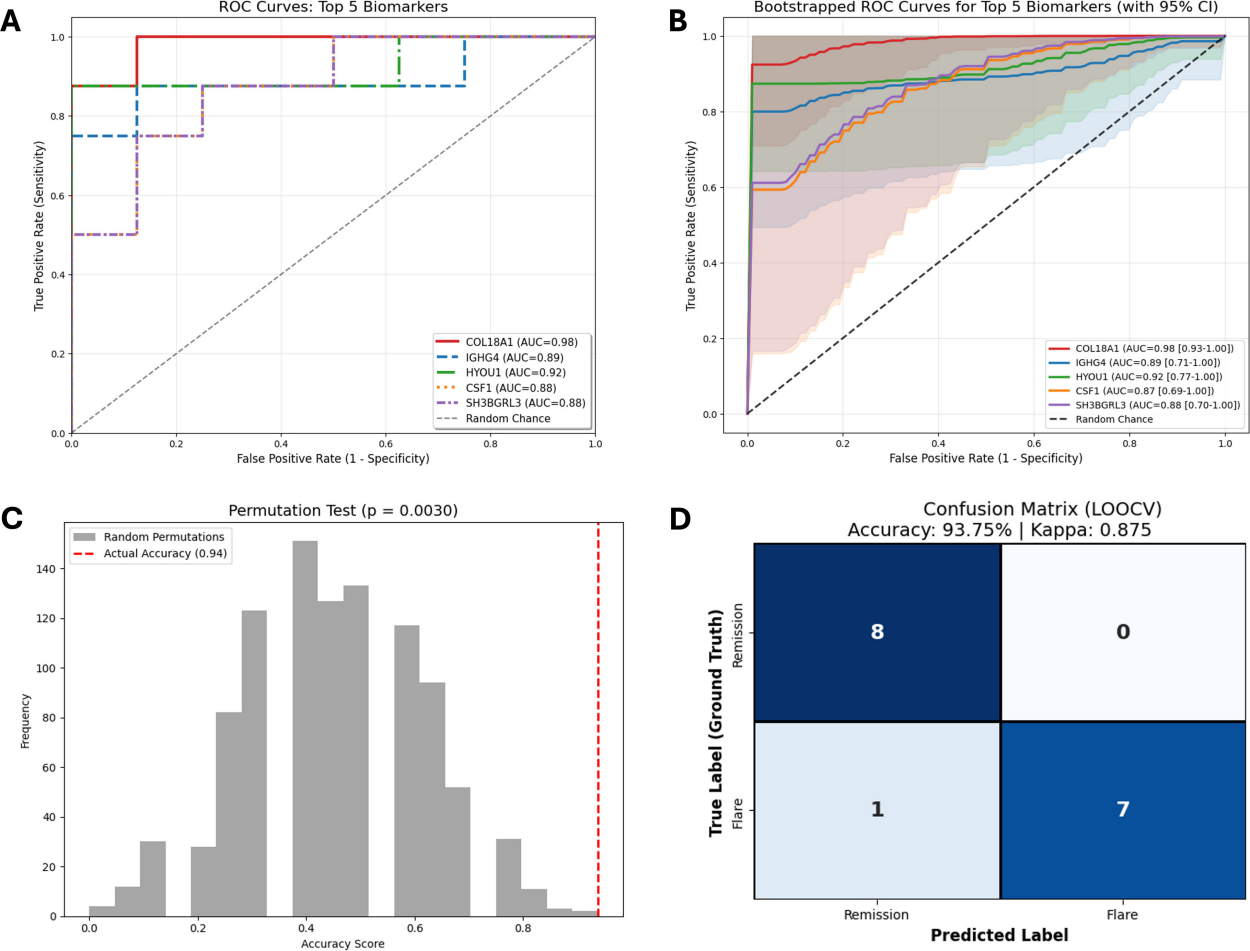
Machine learning validation panel. **(A)** ROC Curves: Top 5 Biomarkers. Individual ROC curves for top biomarkers, such as COL18A1 (AUC = 0.98) and SH3BGRL3 (AUC = 0.88), both outperform random chance. The varied AUCs suggest a hierarchy of biomarker utility, with COL18A1 being the most discriminative. **(B)** Bootstrapped ROC curves (with 95% CI) for top 5 biomarkers, e.g., COL18A1 (AUC = 0.96 [0.93-1.00]) and IGHG4 (0.89 [0.71-1.00]), with 95% CIs, exhibit strong diagnostic efficacy and robustness and endorse COL18A1 for flare identification. Cross-validated Random Forest yielded 93.75% accuracy (15/16), Kappa 0.875, AUROC 0.96 (0.84-1.00), 87.5% sensitivity, 100% specificity, no remission misclassifications (**Table 4**). **(C)** Permutation Test (p = 0.0030). The histogram of accuracy scores from 1,000 random label permutations peaks around 0.5, whereas the true accuracy (0.94) lies far to the right, indicating that the predictions reflect a true underlying biological signal. **(D)** Confusion Matrix (LOOCV). The heatmap displays the classification performance. The model correctly identified 8/8 remission patients (100% Specificity) and 7/8 flare patients (87.5% Sensitivity), with only one flare sample misclassified as remission.

Finally, to integrate these highly correlated individual markers into a cohesive, actionable diagnostic tool, we trained a Random Forest classifier on the optimized 5-marker biosignature. The resulting model demonstrated exceptional predictive robustness and diagnostic efficacy, as confirmed by comprehensive cross-validation and rigorous permutation testing (**Figure 11**, **Table 4**). A comprehensive heatmap of all measured molecules further illustrates this systemic shift (**Supplementary Figure 7**).

While the primary Random Forest model utilized LOOCV to maximize training data utility (**Table 4**), we rigorously assessed the stability of these metrics to rule out optimism bias. A secondary sensitivity analysis employing repeated stratified k-fold cross-validation (4-fold, 50 iterations) was conducted as a computational stress-test. This rigorous secondary validation yielded a conservative mean accuracy of 91.75%, confirming that the high discriminatory performance of the 5-marker biosignature is robust and not an artifact of the Leave-One-Out methodology.

Sensitivity Analysis: To rigorously assess model stability and address the potential for optimism bias in small cohorts, we performed a Repeated Stratified K-Fold Sensitivity Analysis (4-fold cross-validation, repeated 50 times). This ‘stress test,’ which reduced the training set size to 75% per fold (n=12), yielded a conservative mean accuracy of 91.75% and a mean AUROC of 0.97. The minimal performance drop relative to LOOCV (93.75%) confirms that the proteomic signature provides a robust, generalizable signal, even under stricter validation conditions.

## 4 Discussion

The discovery phase proteomic landscape of this South Asian SLE cohort reveals a coordinated reorganization of the circulating proteome during the transition from remission to active flare. While these findings provide high-dimensional insights into disease activity, it is critical to distinguish between molecular associations and functional causality.

### 4.1 Proteomic signatures of lupus flare

Flare samples demonstrate significant upregulation of proteins associated with vascular stress (AGT, COL18A1), epithelial barrier remodeling (CDH1), and acute-phase inflammatory responses (HPR) (**Table 3**, **Figure 2**). Rather than acting as confirmed causal agents that drive the disease process, these proteins serve as correlated surrogates or markers of the underlying endothelial stress and systemic inflammatory responses occurring during a flare.

These molecular alterations show positive correlations with renal, mucocutaneous, and general BILAG domains (**Figure 8**) (12). Pathway enrichment analyses via Reactome and Gene Ontology reinforce these observations, highlighting the activation of extracellular matrix organization (13), integrin-mediated cell surface interactions (14), and scavenger receptor pathways (15) (**Figure 4**). These processes are consistent with systemic immune activation and endothelial dysfunction.

For instance, the robust elevation of COL18A1, which correlated positively with renal and mucocutaneous BILAG domains, likely mirrors the microvascular remodeling and basement membrane changes inherent in active SLE pathology (16, 17). Similarly, the upregulation of Angiotensinogen (AGT) aligns with the systemic inflammatory tone but is interpreted here as a molecular indicator of vascular activation rather than a primary driver of inflammation (18). Beyond its canonical role in blood pressure regulation, angiotensin II can promote pro-inflammatory cytokine production, oxidative stress, and immune cell activation, mechanisms that may contribute to lupus-associated vascular and renal pathology (19). Haptoglobin-related protein (HPR), an acute-phase reactant involved in hemoglobin scavenging, showed elevated expression during flare, likely reflecting heightened hemolysis, oxidative stress, and systemic inflammation characteristic of active disease (20). These findings are consistent with other studies that have observed the activation of inflammatory and complement pathways in active SLE, which are critical components of the disease’s pathophysiology (21).

### 4.2 Proteomic signatures of remission

SLE remission is characterized by the restoration of structural homeostasis and immune quiescence rather than a mere absence of inflammation. Filamin A (FLNA), FERMT3, and Transgelin-2 (TAGLN2) (**Figures 2**, **7**), components of the cytoskeletal machinery essential for immune cell motility, show relative preservation during remission (22). The stabilization of these proteins suggests a restoration of physiological cellular architecture, which supports immune resolution and tissue repair.

Filamin A (FLNA), Ferritin family member 3 (FERMT3/kindlin-3), and Transgelin-2 (TAGLN2) constitute interconnected components of the cytoskeletal machinery essential for immune cell motility, adhesion, and mechanotransduction. Their downregulation during SLE flare, in contrast to relative preservation during remission, may reflect diminished leukocyte trafficking capacity and compromised immune synapse dynamics during active disease. This cytoskeletal reorganization could represent a compensatory mechanism limiting excessive immune cell recruitment and tissue infiltration. Alternatively, it may indicate immune cell exhaustion or functional impairment during sustained inflammatory stress in SLE patients. The relative stabilization of these proteins during remission suggests restoration of physiological cellular architecture, supporting immune resolution and tissue repair processes. Several studies have identified unique proteomic signatures associated with disease activity and specific organ involvement in SLE, demonstrating the utility of proteomic analysis in understanding disease pathogenesis and identifying potential biomarkers (23; 24; 12). Specifically, research has shown distinct protein clusters correlating with renal disease in lupus nephritis, as well as unique signatures in discoid lupus patients, including elevated immunoglobulin A autoantibodies and interleukin-23 (25).

IGHG4 (Immunoglobulin Heavy Constant Gamma 4) downregulation during flare is intriguing, given IgG4’s known anti-inflammatory properties and inability to activate complement. This suppression might reflect a shift toward more pro-inflammatory immunoglobulin subclasses during active disease, although this hypothesis requires validation through immunoglobulin subclass-specific assays (26; 27).

Importantly, stratified correlation analyses revealed residual activity of select proteins (AGT, COL18A1, CDH1) even during clinical remission, suggesting subclinical molecular dysregulation that may precede clinical relapse of lupus (23; 12). This finding aligns with plasma proteomic studies that identify signatures associated with disease activity and organ involvement beyond clinical scores (25; 24), underscoring the potential value of molecular monitoring to detect early signs of an impending flare or incomplete disease control.

The clustering of clinical outliers (ASF_7 and ASR_5) suggests that proteomic shifts, particularly along the HYOU1/COL18A1 axis, may precede changes in clinical Easy-BILAG scores, indicating a ‘molecular lag’ where systemic profiling detects subclinical activity before overt flare manifestations.

### 4.3 Systems-level integration of metabolic and structural networks

The proteomic landscape of Indian SLE patients reveals a coordinated shift in cellular resource allocation during the transition from remission to active flare. Our findings demonstrate that active disease is not merely an increase in inflammatory markers but a fundamental reorganization of the circulating proteome. The significant overrepresentation of translational machinery, specifically components governing ribosomal initiation and elongation, suggests a surge in protein biosynthesis required to sustain activated immune cell populations.

This increased metabolic demand is coupled with a distinct metabolic trade-off. Reactome analysis shows a concurrent suppression of glycolytic and gluconeogenic pathways during flare, likely reflecting immune cell metabolic exhaustion or systemic stress. This bioenergetic shift is structurally anchored by the loss of cytoskeletal integrity. High-connectivity network modules reveal the collective downregulation of FLNA, FERMT3, and TAGLN2. These proteins are essential for leukocyte motility and immune synapse dynamics; their suppression during SLE flare may represent a compensatory mechanism to limit excessive tissue infiltration or, conversely, a marker of functional impairment under sustained inflammatory pressure (28; 29). This intricate interplay between metabolic reprogramming and cytoskeletal restructuring underscores a finely tuned systemic response during active disease, highlighting the complex adaptive strategies employed by the host immune system.

### 4.4 The HYOU1 mechanistic hub: bridging vascular and immune axes

Our integrated network analysis identifies HYOU1 (Hypoxia Up-Regulated 1) as a central hub that orchestrates this proteomic reprogramming (**Figure 9**). While proteins like COL18A1 and HPR indicate acute-phase induction and endothelial stress, and IGHG4 marks the suppression of immunoregulatory circuits, HYOU1 acts as the topological bridge between these disparate pathways. HYOU1 is involved in glucose deficiency-induced metabolic stress, endoplasmic reticulum stress, and immune responses, suggesting its intersection with these processes in disease pathogenesis (30). Its upregulation during active disease, therefore, could reflect an adaptive response to cellular stress, simultaneously modulating protein folding and immune responses within the inflamed microenvironment.

Our network analysis identifies HYOU1 as a central hub, implying it acts as a molecular surrogate where hypoxia-related ER stress converges with vascular injury. Specifically, HYOU1 may coordinate the transition to a flare state by signaling the downregulation of cytoskeletal components like FLNA and FERMT3.

The high centrality of HYOU1 supports a mechanistic model where hypoxia-related endoplasmic reticulum (ER) stress converges with vascular remodeling and humoral immune dysfunction. This significant finding suggests that HYOU1 is a candidate lead of the molecular shifts characterizing SLE flares in the Indian population. Furthermore, the persistence of these molecular signatures in outlier patients who were clinically classified as in remission suggests that this HYOU1-centered axis may detect subclinical molecular dysregulation before clinical symptoms manifest (31).

While network analysis identifies HYOU1 as a central topological hub, we emphasize that network centrality does not imply functional causality. These findings are hypothesis-generating; subsequent functional validation, such as *in vitro* knockdown or animal models, remains a necessary future step to establish definitive causal relationships between these candidate leads and SLE mechanisms.

### 4.5 Metabolic reprogramming in lupus activity

Gene Ontology enrichment analysis revealed significant overrepresentation of components of the translational machinery and protein biosynthesis pathways among differentially expressed proteins (**Figure 4A****;** **Supplementary Figure 5**). Enrichment of terms related to translational initiation, elongation, and termination suggests dynamic modulation of ribosomal activity during SLE disease transitions. This proteomic shift toward enhanced or dysregulated protein synthesis likely reflects the metabolic demands of activated immune cells during lupus activity, consistent with the known metabolic reprogramming that accompanies lymphocyte activation and differentiation (32; 33).

Reactome analysis of downregulated proteins identified suppression of glycolytic and gluconeogenic pathways during a flare (**Figure 4C**), potentially reflecting immune cell metabolic exhaustion or systemic metabolic stress. The simultaneous upregulation of acute-phase proteins and downregulation of metabolic enzymes suggests a coordinated shift in cellular resource allocation prioritizing inflammatory protein production while compromising energy metabolism homeostasis. This metabolic reorganization, characterized by a shift from efficient energy production to rapid biosynthesis, is a hallmark of activated immune responses (34).

### 4.6 Network biology and protein-protein interactions

Protein-protein interaction network analysis revealed tightly interconnected modules among differentially expressed proteins, highlighting coordinated rather than isolated proteomic changes (**Figure 4D**). A prominent cluster comprising cytoskeletal proteins (FLNA, FERMT3, TAGLN2, MSN) showed collective downregulation during flare, reinforcing the concept of systemic cytoskeletal reorganization as a hallmark of active disease. A second cluster of metabolic enzymes (GAPDH, PKM, ENO1) reflected altered glycolytic flux. Central hub proteins involved in stress response and cellular signaling (HSPA8, PDIA3, YWHAZ) showed high connectivity, suggesting their role as coordinators of proteomic reprogramming during disease state transitions. For example, the enrichment of proteins involved in the coagulation cascade and complement system further substantiates the complex interplay between inflammation, immunity, and coagulation in SLE pathogenesis (35).

### 4.7 Cytokines as complementary flare readouts

The PCA analysis and dense co-expression modules indicate that cytokines capture a broader “systemic inflammatory tone” in SLE, with flares characterized by upregulation of chemokine and myeloid growth-factor networks that partially separate from remission in low-dimensional space (**Figure 6**). This pattern is consistent with larger cohort studies, in which interferon-regulated chemokines and myeloid mediators track disease activity but exhibit intra-patient variability and overlap (36).

### 4.8 Biological alignment with known SLE pathways

The prominence of CSF1, CCL2/CCL13, CXCL9, and TSLP in the differential expression profile aligns with current understanding of SLE pathogenesis, in which activated macrophage and monocyte lineages, CCR2-dependent recruitment, and interferon-driven Th1 chemokine axes contribute to tissue damage and flare. The strong intra-module correlations across chemokines and growth factors in the heatmap suggest that the protein panel centered on COL18A1 and HYOU1 is embedded within a broader inflammatory network rather than acting in isolation, reinforcing the argument for multi-marker signatures rather than single-analyte diagnostics. This emphasizes the need for comprehensive biomarker panels that can capture the multifaceted nature of systemic autoimmunity, moving beyond individual inflammatory markers (37).

### 4.9 Translational implications

From a translational perspective, these cytokine findings support using chemokine panels as dynamic activity markers that could complement more structurally anchored proteins in longitudinal monitoring, particularly where rapid shifts in inflammation are expected. However, given the small cohort, cytokine-based discrimination should be regarded as exploratory, and future work in larger, prospectively-followed SLE cohorts will need to test whether adding relevant chemokines meaningfully improves flare prediction beyond the core protein signature and standard serologic markers (38).

### 4.10 Machine learning integration and biomarker performance

The study supports a multi-protein inflammatory signature centered on COL18A1, IGHG4, HYOU1, CSF1, and selected chemokines that robustly discriminates SLE flare from remission despite a small cohort, and aligns with emerging literature on multi-marker panels and hypoxic-vascular pathways in lupus. These findings suggest that integrating these genomic signatures into machine learning algorithms could enhance predictive models for SLE disease activity (39).

### 4.11 Principal findings and clinical relevance

The differential expression analysis highlighted COL18A1, CSF1, and CCL13-related proteins as markedly upregulated in flare, and IGHG4 and SH3BGRL3 as downregulated, with large effect sizes and clear separation on the volcano and violin plots. These findings extend prior proteomic screens in which broader endothelial and extracellular matrix-related signatures were associated with active SLE and lupus nephritis, emphasizing that no single biomarker is sufficient and that composite panels are likely required (24).

The derivation of a diagnostic cut-off for COL18A1 (>21.78 Log2 abundance) yielded near-complete separation between flare and remission (**Figure 7B**). Unlike traditional continuous biomarkers such as anti-dsDNA titers, which often have overlapping “gray zones,” COL18A1 demonstrated a sharp decision boundary with 100% specificity in this cohort. This suggests its potential practical application as a binary ‘rule-in’ tool in the clinic, where values exceeding 21.78 could objectively confirm flare status even in cases with ambiguous clinical presentation. Complementing this, the ROC analyses for the top proteins (including COL18A1 and SH3BGRL3) demonstrated AUC values of approximately 0.9 or higher (**Figures 11A, B**), comparable to or exceeding those of previously proposed flare and renal-flare markers (40).

The proposed biosignature complements existing clinical indices such as Easy-BILAG and SLEDAI by providing molecular granularity. For example, COL18A1 NPX thresholds demonstrated high specificity for flare, suggesting potential as a “rule-in” adjunct to serological markers(anti-dsDNA, complementC3/C4). Similarly, coordinated cytokine modules(CXCL9, CCL2, CCL3, CCL13) may serve as dynamic indicators of flare biology, supporting integration into longitudinal monitoring workflows (41). Further investigation into the Clinical utility of these protein signatures in larger, more diverse cohorts is warranted to validate their predictive accuracy and assess their impact on patient management (23). Integrating these novel proteomic biomarkers into advanced machine learning algorithms could further refine predictive models for SLE activity, enabling more precise and proactive therapeutic interventions (42). This approach, leveraging both proteomic insights and computational analytics, could facilitate the development of stratified treatment strategies tailored to individual patient profiles (28).

### 4.12 Integration with disease activity and pathobiology

Correlation with Easy-BILAG scores revealed that chemokines, such as CCL13, were positively associated with clinical activity. Similarly, CSF1 displayed a strong positive association, indicating coordinated upregulation of myeloid and stromal signals during active disease. These observations are consistent with the literature, which identifies multiple chemokines (e.g., CCL2, CXCL10, CCL19) and macrophage-related mediators as quantitative surrogates of SLE activity and organ involvement. Specifically, CSF1 has been identified as a robust indicator of active lupus nephritis, exhibiting high sensitivity and specificity in distinguishing active from inactive renal disease (43).

Network analysis revealed HYOU1 as a central hub connecting FLNA, SH3BGRL3, IGHG4, COL18A1, and other nodes in both protein-only and integrated protein-cytokine graphs. This supports a mechanistic model in which hypoxia/ER-stress-related chaperones and endothelial-matrix remodeling converge with humoral immune effectors, consistent with reports implicating HYOU1/ER-stress pathways and endothelial injury in SLE and lupus nephritis (44; 45).

### 4.13 Model performance, robustness, and explainability

The Random Forest classifier, trained on preselected biomarkers, achieved robust separation between flare and remission in PCA space. In this n=16 pilot cohort, it achieved 93.75% cross-validated accuracy, a mean AUROC of 0.96, and a Cohen’s Kappa of 0.88 (**Table 4**), metrics comparable to or exceeding those reported in prior proteomic and cytokine ML models for SLE flare prediction and disease activity discrimination in Asian and diverse cohorts (42). Repeated stratified k-fold validation maintained 91.75% accuracy, mitigating Leave-One-Out artifacts and aligning with established ML approaches using lab, transcriptomic, and macrophage-derived data for lupus activity forecasting (39; 38).

By utilizing SHAP interpretability analysis, we identified COL18A1 as the primary predictor of flare states (**Figure 10B**). In this context, the derived abundance threshold for COL18A1 (>21.78 Log2) functions as an objective “rule-in” tool to confirm active disease. We emphasize that these markers act as biological readouts of the current disease state; their stability and discriminative power endorse their potential for longitudinal monitoring to detect subclinical molecular activity before overt clinical manifestations occur.

The confusion matrix showed one false negative and zero false positives (Kappa = 0.88), underscoring the high reliability of positive flare detection (**Figure 11D**). COL18A1 and IGHG4 emerge as compelling hypothesis-generating leads. While these small-cohort results are promising, larger, heterogeneous validation studies are essential to confirm generalizability (23).

### 4.14 Novel biomarker candidates

This study identifies novel proteins as potential SLE biomarkers, including WDR1 (actin polymerization regulator) and PROF1 (actin-binding protein), which show differential expression implicating cytoskeletal remodeling in immune cells. Their consistent dysregulation and network connectivity warrant further validation as candidates. Such investigations could reveal their precise roles in SLE pathogenesis and potential utility in diagnostic or prognostic panels.

Methodologically, hypothesis-free proteomics, integrated with organ-stratified BILAG scoring, reveals domain-specific correlations, such as AGT with renal involvement (46; 25; 24). PCA achieves robust separation between SLE flares and remissions (PC1: 56.7% variance), with outliers suggesting transitional states, highlighting proteomics’ sensitivity.

Addressing gaps in Indian SLE cohorts, findings account for ancestry-specific genetics (HLA, complement, FCGR polymorphisms) and environmental factors (infections, diet, vitamin D deficiency). Translational potential includes ELISA/multiplex assays for monitoring in resource-limited settings. Although these overlap with known markers (acute-phase proteins), novel genes such as COL18A1, CDH1, FERMT3, and TAGLN2 merit deeper investigation. Further studies should focus on validating these markers in larger, diverse populations to confirm their consistency across varying genetic and environmental backgrounds. The identified protein-based models and the combined clinical-proteomic models demonstrate superior predictive accuracy, suggesting their utility for identifying novel molecular pathways associated with SLE flares (28).

### 4.15 Integration of clinical and molecular stratification

A key strength of this study lies in integrating unbiased proteomic profiling with validated organ-specific assessment of SLE disease activity. Domain-wise correlation analyses revealed protein-phenotype associations with specific BILAG domains, suggesting that certain proteins may serve as targeted biomarkers for particular organ manifestations. For example, positive correlations of AGT, COL18A1, and HPR with renal and mucocutaneous domains indicate their potential relevance for monitoring lupus nephritis and cutaneous manifestations, respectively.

Principal Component Analysis demonstrated robust separation between the SLE flare and remission groups along the primary axis of variance (PC1: 56.7%), validating the discriminative power of the proteomic stratification (**Figure 5**). Notably, the observation of specific sample-level outliers, such as ASF_7 clustering with the remission cohort and ASR_5 clustering with the flare cohort, highlights a critical translational advantage of molecular profiling over conventional clinical assessment. Rather than analytical noise, these cases likely represent transitional disease states characterized by subclinical molecular activity that evades detection by standard clinical scoring. This discordance suggests that proteomic reprogramming, specifically along the HYOU1/COL18A1 axis, may precede the overt clinical manifestations captured by the Easy-BILAG instrument. Recognizing this ‘molecular lag’ underscores the enhanced sensitivity of systemic profiling to detect subtle disease dynamics, thereby identifying a crucial therapeutic window for early SLE flare prediction and intervention before irreversible organ damage occurs (47). Proactive identification of impending flares through advanced proteomic analysis could significantly improve patient outcomes by enabling timely, targeted therapeutic adjustments (47).

### 4.16 Global representation and population-specific considerations

Our study addresses a critical gap in global lupus research by examining an Indian (South Asian) cohort, a population historically underrepresented in proteomic investigations, which has been predominantly studied in prior research on European or East Asian ancestries (48). Genetic polymorphisms prevalent in South Asian populations, such as variations in HLA alleles and complement components, may distinctly influence molecular signatures. Building on limited prior efforts in Eastern Indian cohorts using cytokines and machine learning (38), this represents the first integrated plasma proteomic-ML analysis in a South Asian SLE population. While these findings offer preliminary insights into the Indian SLE population, the observed expression patterns of COL18A1 and IGHG4 warrant rigorous multi-ethnic validation to ensure broad clinical translatability.

### 4.17 Clinical translation potential

The candidate biomarkers identified in this SLE study hold considerable potential for translation into clinical practice, especially in resource-limited settings common across India. Proteins such as AGT, COL18A1, CDH1, and HPR, which showed strong associations with disease activity, could be developed into cost-effective ELISA-based panels or targeted mass spectrometry assays suitable for deployment in rheumatology clinics. Such tools would enable:

- longitudinal disease monitoring with objective molecular readouts complementing clinical assessments;
- early detection of subclinical SLE disease activity potentially preceding clinical flares;
- personalized therapeutic stratification based on individual proteomic profiles;
- treatment response monitoring with molecular endpoints.

Although several identified proteins overlap with previously reported lupus biomarkers (49; 50), candidates such as COL18A1, CDH1, and FERMT3 represent novel additions with limited prior documentation in the comprehensive SLE proteomic atlas, warranting deeper mechanistic investigations into their roles in endothelial activation, hypoxic stress, and flare transitions.

### 4.18 Limitations

While this investigation establishes a novel molecular framework for the precision stratification of SLE activity in an underrepresented South Asian cohort, several constraints must be acknowledged to contextualize the findings. The primary limitation is the modest cohort of 16 patients (8 per group), which positions this work as a discovery-phase pilot study rather than a definitive biomarker validation effort (38). Although the sample size restricts global generalizability, we implemented robust computational safeguards, including LOOCV and 1,000-fold permutation testing, to ensure the biological signal significantly exceeds random chance (p=0.0030). Consequently, the identified five-protein signature (COL18A1, HYOU1, IGHG4, FLNA, and SH3BGRL3) is strictly characterized as a set of “candidate leads”. These markers effectively capture the multifactorial pathophysiology of SLE flares within this specific cohort, but they require independent, high-powered validation in larger, multi-centric studies to establish clinical reliability and ancestry-specific utility.

Furthermore, the current cross-sectional architecture enables robust stratification of flare versus remission but precludes the assessment of temporal molecular dynamics. Longitudinal follow-up is essential to determine whether these leads can predict intra-individual disease-state transitions or identify subclinical “molecular lags” that evade detection by standard clinical instruments such as the Easy-BILAG (51). Despite the cohorts being demographically matched, patients presented with heterogeneous clinical manifestations and variable immunosuppressive regimens. While medication exposure (including corticosteroids and mycophenolate mofetil) was statistically comparable between groups, the potential for treatment-induced proteomic variation, particularly regarding immunoregulatory markers such as IGHG4, cannot be entirely excluded without subgroup analysis of drug-naïve patients.

Finally, the proposed AI-driven classification model serves as an exploratory framework designed to enhance objective disease monitoring. Further refinement of diagnostic thresholds, such as the COL18A1 NPX cut-off, is required to transition from a pilot “rule-in” tool to a clinically applicable diagnostic assay. While integrating plasma proteomics and cytokine profiling provided deep biological insights, the absence of complementary multi-omics layers, such as transcriptomics or epigenomics, limits the resolution of the full causal pathways underlying the identified vascular-endothelial stress signature (48). These findings should thus be interpreted as a preliminary foundation for future precision medicine initiatives in South Asian SLE populations (28; 38).

### 4.19 Future directions

The preliminary findings of this discovery-phase pilot study justify expanded longitudinal efforts to translate these molecular signals into clinically actionable tools. Future multi-center studies across diverse regions of India are essential to validate these observations and address key limitations, building on prior Eastern Indian cytokine-ML research (38). These studies should incorporate the following priorities:

- Larger, independent validation cohorts (n > 100) with strict statistical controls, including multiple-testing corrections (e.g., FDR < 0.05), to enhance statistical power and detect low-abundance markers.
- Longitudinal sampling capturing intra-individual disease state transitions, including prospective monitoring of subclinical flares via serial Easy-BILAG assessments coupled with proteomic profiling. This will establish the predictive utility of the HYOU1/COL18A1 axis and determine if these markers can detect subclinical “molecular lags” before overt clinical flares occur.
- Treatment-stratified analyses (e.g., propensity score matching or drug-naïve subgroups) to definitively distinguish intrinsic disease-related proteomic changes from therapy-induced effects, such as corticosteroid modulation of IGHG4 or SH3BGRL3.
- Multi-ethnic validation across broader South Asian ancestries and global cohorts to assess the generalizability of the 5-protein biosignature, identify population-specific variations (e.g., ancestry-modulated COL18A1), and benchmark against established Western proteomic atlases.
- Functional mechanistic studies, including *in vitro* knockdown/overexpression and animal models, to establish causal relationships between novel candidates (e.g., COL18A1, HYOU1) and SLE mechanisms such as endothelial activation and hypoxic stress.

Additionally, integration of complementary multi-omics platforms (genomics, epigenomics, single-cell transcriptomics) would provide a more comprehensive systems-biology portrait of lupus endotypes. Correlation with long-term clinical outcomes (e.g., flare prediction, organ damage via SLICC-ACR/SDI) would rigorously establish the prognostic and predictive value of the 5-protein biosignature, paving the way for clinical decision-support tools in resource-limited settings.

## 5 Conclusion

This investigation provides a robust molecular framework for the precision stratification of Systemic Lupus Erythematosus (SLE) activity within an underrepresented South Asian cohort. By integrating LC-MS/MS-based plasma proteomics with multiplexed cytokine profiling, we have delineated a fundamental molecular polarization between clinical states. Our findings establish that active exacerbation is not merely a quantitative increase in inflammation, but a qualitative shift toward endothelial activation and hypoxic stress, characterized by the coordinated induction of the COL18A1 and HYOU1 axes. Conversely, clinical remission is defined by the active maintenance of cytoskeletal integrity and immunoregulatory homeostasis, specifically through the preservation of FLNA, SH3BGRL3, and IGHG4.

Using an AI-driven feature selection pipeline, we reduced these high-dimensional data to a parsimonious 5-protein biosignature of candidate leads as a hypothesis-generating foundation for a larger longitudinal validation to predict lupus flare. Validated via a Random Forest ensemble classifier, this signature achieved superior diagnostic performance, yielding a cross-validated accuracy of 93.75% and a mean AUROC of 0.96. Critically, establishing a COL18A1 NPX threshold demonstrated 100% specificity, positioning it as a potentially transformative “rule-in” tool for confirming active disease in clinically ambiguous presentations.

From a systems-biology perspective, the identification of HYOU1 as a central topological hub connects hypoxia/ER-stress-related chaperones with endothelial remodeling and humoral effectors, offering new mechanistic insights into the multifaceted pathophysiology of SLE flares. While this pilot study establishes a reproducible pipeline for precision activity assessment, these findings mandate rigorous external validation in larger, longitudinal, and multi-ancestry cohorts to confirm their clinical utility. Ultimately, this work addresses a critical knowledge deficit in the global biomarker evidence base and advocates for a transition toward integrative, multi-platform molecular signatures for the management of systemic autoimmunity.

## Data Availability

The original contributions presented in the study are included in the article/**Supplementary Material**. Further inquiries can be directed to the corresponding authors.
