# Supplementary material for: Integrative plasma proteomics and myeloid- interferon profiling reveal an AI-validated vascular- endothelial stress signature distinguishing SLE flare from remission in an Indian cohort a discovery – phase study
Source: Front Immunol. 2026 Jun 3;17:1819049. doi: 10.3389/fimmu.2026.1819049 (PMC13272295; doi:10.3389/fimmu.2026.1819049)
Supplement: Supplementary file 1 [file Table1.docx]

**Methodology (Supplementary):**

Spearman correlation analyses were performed using log_2_-transformed, normalised LFQ intensities. PCA was conducted using centered and scaled protein abundance values. STRING network analysis was performed using default confidence thresholds, with interaction sources including experimental data, curated databases, and co-expression. Reactome and Gene Ontology enrichment analyses were conducted using over-representation analysis with a nominal p-value threshold of 0.05. Due to the discovery-phase nature of this pilot study (n=16), top-ranked markers were identified using a nominal p-value threshold (p < 0.05) to prioritize biological signal for subsequent AI-based validation.

Domain-wise correlation analysis revealed distinct protein–phenotype associations across Easy-BILAG domains. Proteins including AGT, COL18A1, CDH1, and HPR showed positive correlations with mucocutaneous, renal, and general domains, suggesting their involvement in vascular stress, extracellular matrix remodelling, and systemic inflammation during flare. In contrast, proteins such as FLNA, FERMT3, TAGLN2, and MSN were negatively correlated with musculoskeletal and haematological domains, potentially reflecting cytoskeletal integrity and immune regulation in remission. Unsupervised hierarchical clustering identified coherent protein modules with domain-specific relevance, supporting their utility as targeted biomarkers for stratifying organ-level disease activity in lupus. These cytoskeletal proteins are integral to immune cell motility and adhesion; their reduced abundance during flare and relative preservation in remission suggest a role in stabilizing cellular architecture and supporting immune resolution.

Stratified correlation analysis further revealed distinct protein-activity relationships across flare and remission states. Proteins, including AGT, COL18A1, CDH1, HPR, and AMBP, showed weak or negative correlations with total easy-BILAG score during flare but showed stronger positive correlations in remission, suggesting differential regulation of these pathways across disease states. In contrast, cytoskeletal proteins such as FLNA, FERMT3, and TAGLN2 exhibited negative correlations with both flare and remission states, with stronger negative associations during flare. These patterns suggest that disruption of cytoskeletal integrity and immune cell motility is more pronounced during active disease. Hierarchical clustering identified coherent protein modules with state-specific relevance, reinforcing their potential as dynamic biomarkers for monitoring lupus activity.

Comparative correlation analysis revealed distinct proteomic signatures between flare and remission states. Flare was characterized by positive correlations of vascular and extracellular matrix proteins (AGT, COL18A1, CDH1, HPR) with disease activity, consistent with systemic inflammation, vascular stress, and tissue remodelling. Conversely, cytoskeletal and immune-regulatory proteins (FLNA, FERMT3, TAGLN2, IGHG4) were negatively correlated, indicating suppression during active disease. In remission, these negative correlations became more pronounced, particularly for FLNA, FERMT3, and TAGLN2, suggesting restoration of cytoskeletal integrity and immune homeostasis. Residual positive correlations for AGT, COL18A1, and CDH1 highlight subclinical vascular or epithelial activity even in clinically quiescent states. Together, these findings underscore flare as a state of proteomic amplification and remodelling, while remission reflects suppression and stabilization, with select proteins serving as potential longitudinal biomarkers for disease monitoring.

| **Protein** | **Spearman_rho** | **p_value** | **FDR_p** |
| --- | --- | --- | --- |
| WDR1 | -0.346417753482864 | 0.188706152870871 | 0.207576768157958 |
| HPR | 0.324450141287063 | 0.220186043862507 | 0.220186043862507 |
| AGT | 0.504822452725028 | 0.0461114328389129 | 0.131415287822054 |
| IGHG4 | -0.487057439725907 | 0.0556923046699683 | 0.131415287822054 |
| AMBP | 0.5229721455449 | 0.037652358464644 | 0.131415287822054 |
| GAPDH | -0.385191948559983 | 0.140658452442934 | 0.182028585514385 |
| ENO1 | -0.365932351131984 | 0.163345573612538 | 0.19964458997088 |
| PFN1 | -0.326895945707462 | 0.216525723895691 | 0.220186043862507 |
| HSPA8 | -0.349635768692908 | 0.184356905209935 | 0.207576768157958 |
| CDH1 | 0.473733679976566 | 0.0637885963881748 | 0.131415287822054 |
| PKM | -0.436723236228397 | 0.0907695857873204 | 0.142637920522932 |
| FLNA | -0.543313314223124 | 0.0296244152699633 | 0.131415287822054 |
| MSN | -0.473733679976566 | 0.063788596388175 | 0.131415287822054 |
| PDIA3 | -0.505934809358593 | 0.045555513178218 | 0.131415287822054 |
| CORO1A | -0.415869201557766 | 0.109133741301166 | 0.160062820575044 |
| TAGLN2 | -0.395246916173567 | 0.129713893115173 | 0.178356603033363 |
| COL18A1 | 0.689874671465874 | 0.00310300480250474 | 0.0467184477795282 |
| YWHAZ | -0.461890337977152 | 0.0716810660847569 | 0.131415287822054 |
| MAN2A1 | 0.673345156232739 | 0.00424713161632074 | 0.0467184477795282 |
| FERMT3 | -0.535959773130597 | 0.0323591657509953 | 0.131415287822054 |
| SH3BGRL3 | -0.462230338271979 | 0.0714450574493349 | 0.131415287822054 |
| HYOU1 | 0.451527413727665 | 0.0791493039894783 | 0.133944975982194 |

**Supplementary Table 1: Correlation statistics between Plasma Proteins and Total Easy-BILAG score**

Supplementary Table 1 presents Spearman rank correlation coefficients, nominal p-value, and false discovery rate (FDR)-adjusted p-values for associations between plasma protein abundance and Total Easy-BILAG score across the study cohort. Correlation analyses were performed using log_2_-transformed, normalized protein abundance values to assess monotonic relationships between measurements and global disease activity. All reported p-values are two-sided, and FDR correction was applied using the Benjamini-Hochberg method to control for multiple comparisons.


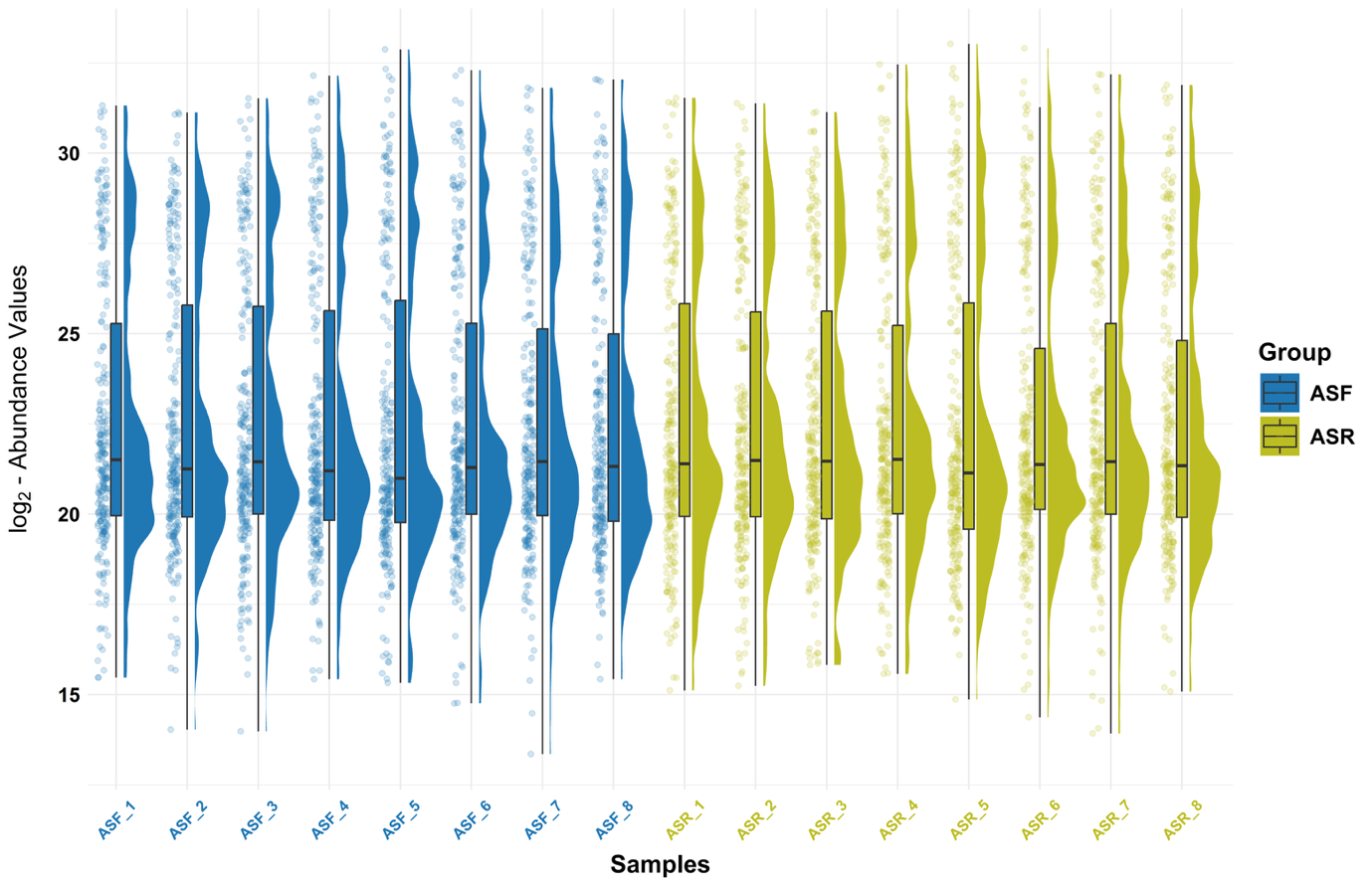


**Supplementary Figure 1: Violin and box plots of log₂-transformed protein abundance across flare and remission samples.**

Distribution of log₂-transformed protein abundance values across individual samples from flare (ASF, blue) and remission (ASR, yellow-green) groups. Each violin plot illustrates the density and spread of abundance values per sample, overlaid with a box plot showing the median and interquartile range. Individual data points are plotted within each distribution. Flare samples exhibit broader distributions and higher median abundances in several cases, consistent with increased proteomic activity during disease flares.


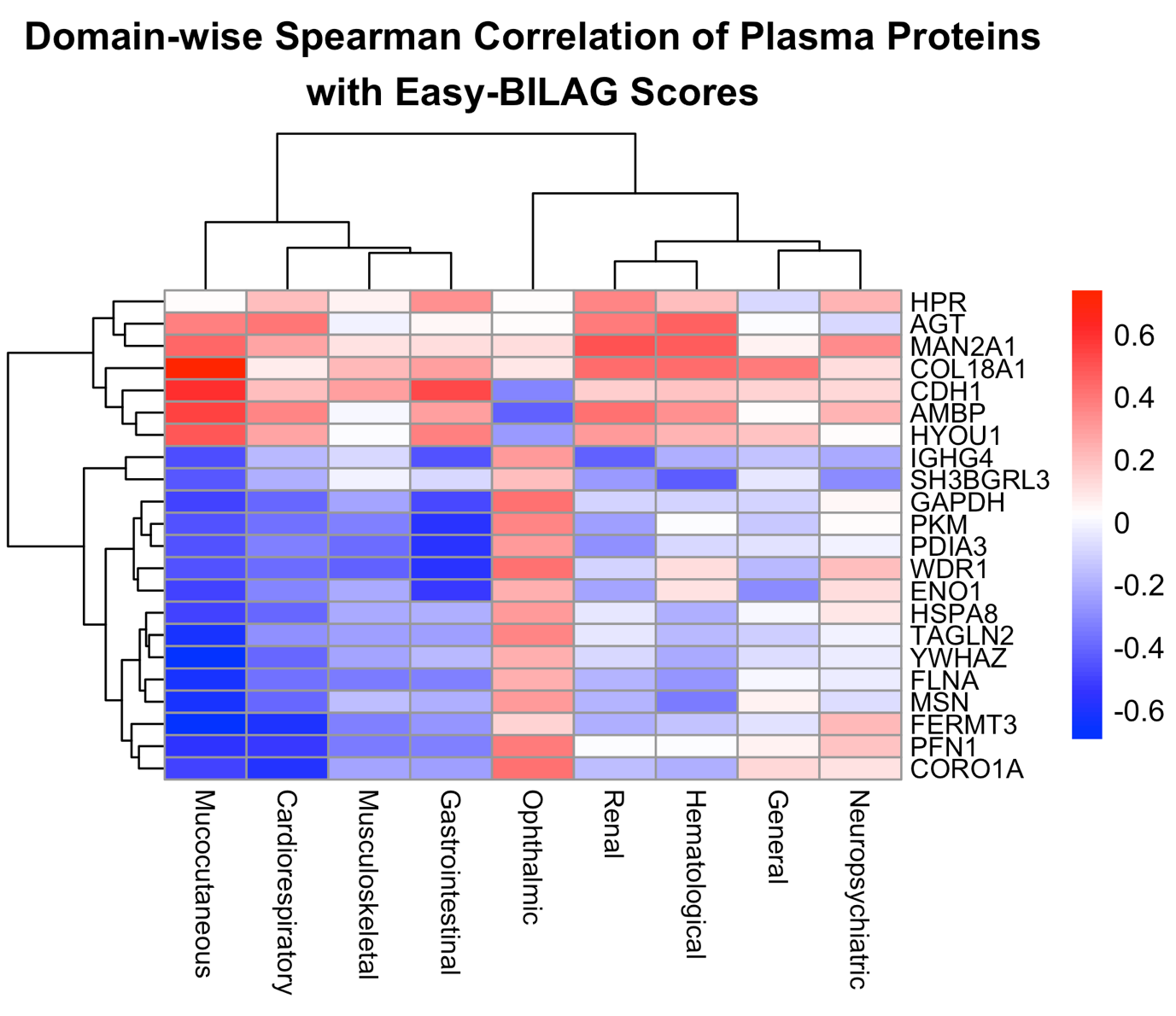


**Supplementary Figure 2: Domain-wise Spearman correlation of plasma protein abundance with Easy-BILAG scores.**

Heatmap displays Spearman correlation coefficients between individual plasma proteins and disease activity scores across nine Easy-BILAG domains. Red indicates a positive correlation, blue indicates a negative correlation, and intensity reflects strength. Unsupervised hierarchical clustering of proteins and domains reveals protein modules with domain-specific correlation patterns. Proteins such as AGT, COL18A1, CDH1, and HPR show positive associations with mucocutaneous, renal, and general domains, while FLNA, FERMT3, and TAGLN2 exhibit negative correlations with musculoskeletal and hematological domains.


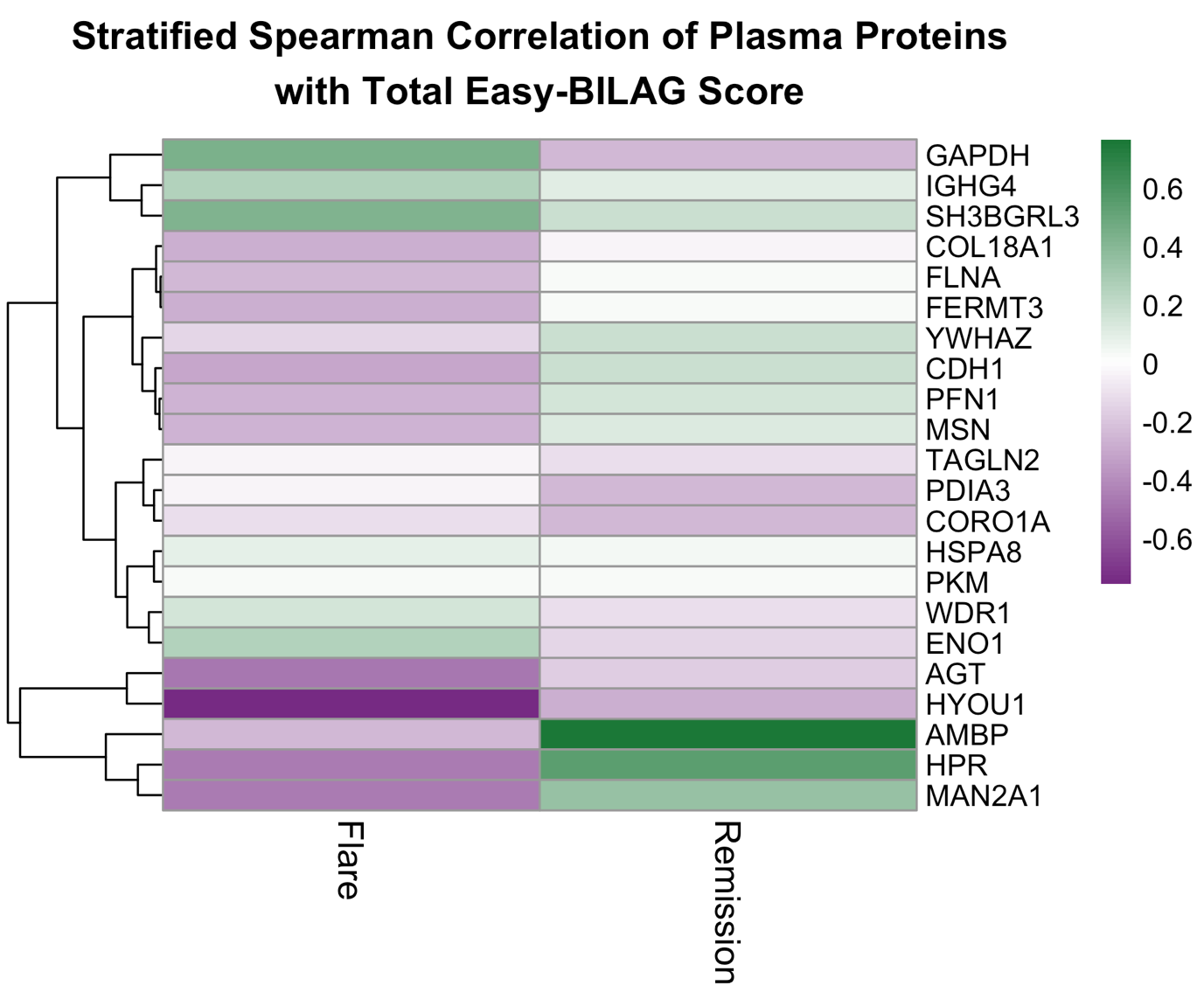


**Supplementary Figure 3: Stratified Spearman correlation of plasma protein abundance with Total Easy-BILAG Score across flare and remission states.**

Heatmap displays Spearman correlation coefficients between individual plasma proteins and overall disease activity measured by Easy-BILAG scores, stratified by flare and remission states. Green indicates positive correlation, purple indicates negative correlation, with intensity reflecting strength. Unsupervised hierarchical clustering reveals differential correlation patterns across disease states. Proteins such as AGT, HPR, AMBP and MAN2A1 show stronger positive correlations with disease activity in remission compared with flare, while cytoskeletal and immune associated proteins such as FLNA, FERMT3, and TAGLN2 exhibit negative correlations across both states with greater magnitude in flare.


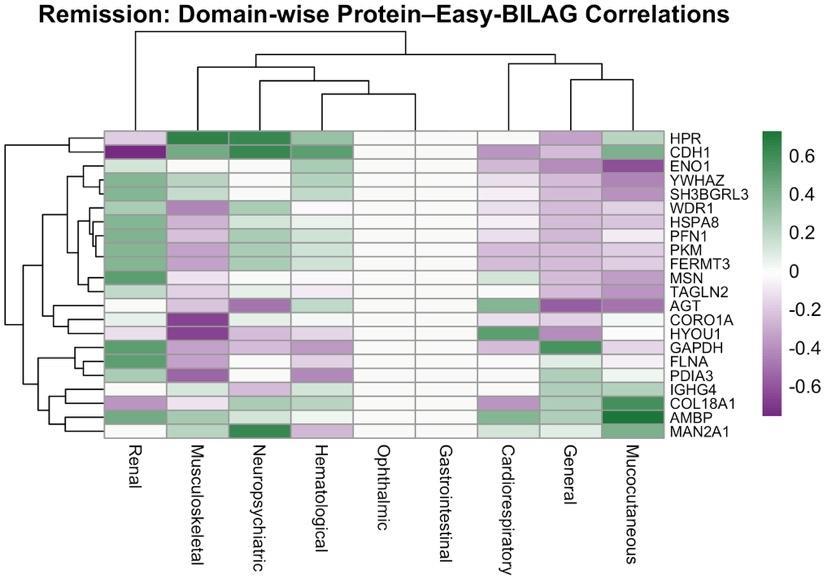

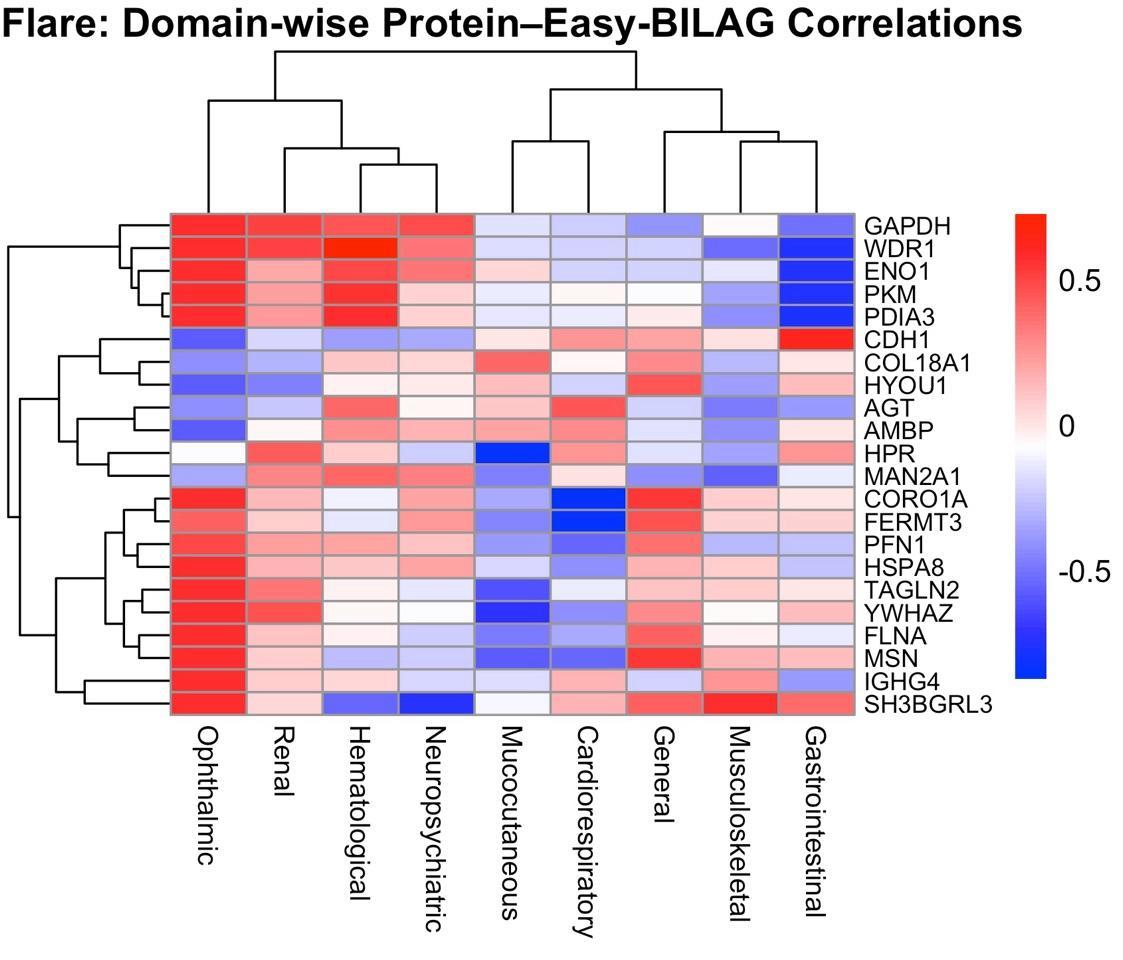


**Supplementary Figure 4: Flare vs. remission domain-specific protein correlations.**

Heatmap comparison of plasma protein-domain associations across Easy-BILAG categories. Flare is characterized by positive correlations of vascular/extracellular matrix proteins (AGT, COL18A1, CDH1, HPR) with renal, mucocutaneous, and general domains, alongside negative correlations of cytoskeletal proteins (FLNA, FERMT3, TAGLN2, MSN) with musculoskeletal and hematological domains. In remission, cytoskeletal proteins remain negatively correlated, reflecting stability and immune regulation, while vascular proteins retain mild positive associations with mucocutaneous and renal domains. These patterns highlight proteomic modules with domain-specific relevance for stratifying lupus activity.


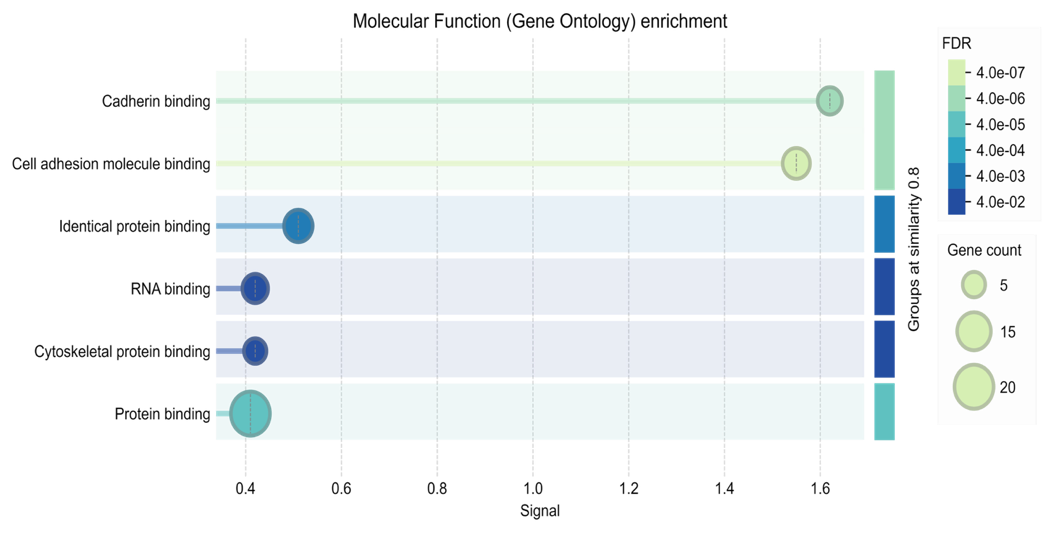


**Supplementary Figure 5:** GO analysis revealed enrichment of translation-related processes, suggesting modulation of ribosomal activity and protein synthesis during lupus flare. These findings point to a proteomic shift toward enhanced or dysregulated protein production, consistent with inflammatory remodeling.

| Cytokine Markers | 1F | 2F | 3F | 4F | 5F | 6F | 7F | 8F |
| --- | --- | --- | --- | --- | --- | --- | --- | --- |
| **CCL8** | 12.18161 | 11.70231 | 11.84791 | 13.21465 | 10.91641 | 13.16534 | 13.49638 | 10.76110 |
| **IL33** | 0.63446 | 0.73214 | 1.22543 | 0.72209 | 0.97731 | 2.16185 | 1.76088 | 0.59963 |
| **CXCL12** | 1.54580 | 1.53170 | 1.27466 | 3.10007 | 3.30527 | 3.07234 | 4.45470 | 3.10999 |
| **OLR1** | 10.03908 | 9.15975 | 9.25912 | 8.24473 | 9.48071 | 8.93315 | 8.13209 | 8.73618 |
| **IL27** | 5.62562 | 3.06917 | 6.10091 | 4.80472 | 6.46757 | 4.80457 | 6.59351 | 4.34006 |
| **IL2** | 0.48943 | 0.47533 | 0.21829 | 0.34678 | -0.04654 | 0.17365 | 0.69014 | 0.08583 |
| **CXCL9** | 10.74332 | 9.88048 | 11.17059 | 10.96030 | 9.67755 | 9.29616 | 12.60812 | 8.82648 |
| **TGFA** | -0.20753 | -0.20256 | 0.58539 | -0.36287 | 0.43737 | -0.29747 | 1.39928 | -0.48455 |
| **IL1B** | -0.28637 | -0.07969 | 0.84410 | 0.46182 | -0.48408 | -0.25942 | 0.64549 | -0.68997 |
| **IL6** | 5.17921 | 6.71175 | 6.94538 | 6.54584 | 5.20623 | 6.98160 | 7.27154 | 4.88735 |
| **IL4** | No Data | No Data | No Data | 2.26310 | 1.86978 | No Data | 2.07260 | 2.00215 |
| **TNFSF12** | 9.87581 | 9.29669 | 9.01380 | 9.69857 | 10.85601 | 10.35059 | 10.77996 | 10.45850 |
| **TSLP** | 0.69617 | 1.17240 | 0.92503 | 0.55352 | 0.16020 | 0.38039 | 0.48220 | 0.29257 |
| **CCL11** | 11.81675 | 12.13335 | 9.94167 | 9.09357 | 11.12118 | 9.80631 | 11.41466 | 9.06455 |
| **HGF** | 9.87300 | 9.99522 | 9.99762 | 11.01779 | 11.79652 | 11.88737 | 12.07856 | 11.70173 |
| **FLT3LG** | 10.29553 | 10.67477 | 10.48255 | 10.12960 | 10.46462 | 10.46858 | 10.70569 | 9.28577 |
| **IL17F** | -2.04691 | -1.50247 | -1.10533 | -1.03072 | -1.14357 | -1.22455 | -1.14542 | -0.67317 |
| **IL7** | -0.01844 | -0.01888 | 1.89240 | 2.59682 | 0.45834 | 3.80089 | 2.39323 | 1.87287 |
| **IL13** | 0.16669 | 0.15259 | -0.10445 | 0.02404 | -0.36928 | -0.14909 | -0.16646 | -0.23691 |
| **IL18** | 11.57763 | 10.33728 | 11.63385 | 12.09740 | 11.90686 | 12.56992 | 12.72472 | 11.10089 |
| **CCL13** | 14.44994 | 15.69339 | 12.84670 | 12.73012 | 13.62286 | 13.70505 | 14.40152 | 12.31255 |
| **TNFSF10** | 9.67497 | 9.13500 | 9.95719 | 9.97384 | 9.42259 | 9.48771 | 10.02584 | 9.28372 |
| **CXCL10** | 11.99586 | 12.22475 | 14.94188 | 14.59167 | 11.91967 | 12.92025 | 14.49137 | 10.92036 |
| **IFNG** | 4.58988 | 3.85358 | 4.85933 | 4.50753 | 2.61883 | 4.44702 | 4.96608 | 3.46823 |
| **IL10** | 1.96488 | 1.83278 | 3.45328 | 2.65867 | 0.67559 | 2.71604 | 4.49852 | 0.11120 |
| **CCL19** | 13.18523 | 13.38077 | 14.54358 | 16.27617 | 16.09607 | 15.54777 | 13.60998 | 14.46468 |
| **TNF** | 2.44487 | 1.21477 | 4.31396 | 3.05546 | 2.14858 | 2.73246 | 3.46947 | 1.45203 |
| **IL15** | 3.98787 | 4.22044 | 4.52617 | 3.60247 | 3.55261 | 4.15990 | 4.03250 | 3.03865 |
| **CCL3** | 9.47145 | 9.02252 | 11.00413 | 12.54519 | 9.40863 | 11.02125 | 10.91527 | 8.03707 |
| **CXCL8** | 8.92916 | 9.32277 | 10.49947 | 9.37859 | 9.37001 | 9.71525 | 9.99615 | 8.07007 |
| **MMP12** | 9.30681 | 7.99134 | 8.10024 | 9.22099 | 9.28270 | 8.57311 | 10.34965 | 8.24508 |
| **CSF2** | 4.21333 | 3.96230 | 4.62940 | 4.31602 | 4.26281 | 4.26106 | 5.47567 | 3.57280 |
| **CSF3** | 4.41335 | 5.85122 | 6.11664 | 5.30560 | 5.06173 | 5.65407 | 6.78650 | 4.51983 |
| **VEGFA** | 12.42809 | 12.51241 | 13.72300 | 13.14646 | 12.88924 | 13.23821 | 15.00763 | 12.41551 |
| **IL17C** | 4.51079 | 4.42458 | 6.03021 | 5.10911 | 5.04851 | 6.63241 | 7.29877 | 4.42862 |
| **EGF** | 7.26676 | 6.76015 | 4.33106 | 9.35814 | 7.13636 | 11.02509 | 7.75462 | 8.72648 |
| **CCL2** | 13.90830 | 14.42441 | 15.82626 | 13.77713 | 13.35361 | 14.09125 | 14.32793 | 12.22344 |
| **IL17A** | -3.36246 | -2.55251 | -2.65108 | -2.77785 | -3.34973 | -2.18269 | -2.35940 | -3.50520 |
| **OSM** | 3.38047 | 3.52431 | 1.72893 | 2.73588 | 4.54566 | 4.85302 | 4.05233 | 5.06923 |
| **CSF1** | 11.95985 | 11.51493 | 12.50735 | 11.62675 | 12.24978 | 11.99135 | 12.47994 | 11.39230 |
| **CCL4** | 10.46238 | 9.83263 | 9.45317 | 12.20162 | 10.61513 | 12.00723 | 10.25420 | 9.00581 |
| **CXCL11** | 14.55346 | 13.89646 | 11.20804 | 12.52695 | 10.06448 | 11.42718 | 12.45629 | 10.39944 |
| **LTA** | 6.27146 | 6.05448 | 6.06267 | 7.81332 | 5.72449 | 7.06000 | 6.43007 | 6.12329 |
| **CCL7** | 7.31225 | 6.86090 | 10.36114 | 5.39867 | 5.52336 | 6.17672 | 7.15340 | 3.04783 |
| **MMP1** | 12.07404 | 10.47985 | 9.53643 | 13.33756 | 10.93820 | 12.39466 | 13.25963 | 10.24248 |

| Cytokine Markers | ASR09 | ASF10 | ASR06 | 1R | 2R | 3R | 4R | 5R |
| --- | --- | --- | --- | --- | --- | --- | --- | --- |
| **CCL8** | 11.69598 | 11.31320 | 14.57490 | 10.61702 | 9.16275 | 12.18089 | 11.08637 | 8.69416 |
| **IL33** | 2.89386 | 2.24009 | 2.97009 | 0.32844 | 0.80636 | 0.95195 | 0.95098 | 0.76667 |
| **CXCL12** | 4.73551 | 4.41193 | 5.14149 | 1.48337 | 1.38185 | 2.24538 | 2.62596 | 3.13614 |
| **OLR1** | 8.97577 | 8.25463 | 9.36602 | 8.09982 | 8.04587 | 7.31645 | 8.49393 | 8.70933 |
| **IL27** | 7.50099 | 9.34996 | 8.63254 | 4.22977 | 4.55852 | 5.55486 | 5.35711 | 4.13448 |
| **IL2** | 2.79800 | 2.83972 | 3.90603 | -0.05293 | -0.04845 | -0.06211 | -0.02918 | 0.01706 |
| **CXCL9** | 10.26549 | 8.60673 | 10.65322 | 7.73566 | 9.54181 | 9.37863 | 8.02981 | 8.28184 |
| **TGFA** | 3.19032 | 2.81016 | 3.46464 | -0.74992 | -1.03255 | -0.76134 | -0.31893 | -1.19661 |
| **IL1B** | 2.65785 | 2.31555 | 5.30096 | -0.82873 | -0.42966 | -0.69571 | 0.66359 | 0.41455 |
| **IL6** | 8.62850 | 8.75119 | 10.56966 | 4.37284 | 6.11855 | 4.40811 | 5.03445 | 5.37027 |
| **IL4** | 4.95719 | 4.80819 | 5.42877 | 1.86339 | No Data | 1.85421 | No Data | No Data |
| **TNFSF12** | 10.20861 | 10.21979 | 10.56078 | 9.75104 | 9.24128 | 9.72114 | 10.27266 | 10.15482 |
| **TSLP** | 0.15501 | -0.21631 | 1.18960 | 0.15381 | 0.15829 | 0.14463 | 0.17756 | 0.30298 |
| **CCL11** | 10.51847 | 8.77734 | 10.36488 | 9.24493 | 9.64026 | 10.31830 | 9.29121 | 9.43074 |
| **HGF** | 12.31984 | 10.65167 | 12.12985 | 10.01183 | 9.88513 | 10.61056 | 11.63494 | 11.22986 |
| **FLT3LG** | 10.23005 | 10.14115 | 10.59739 | 9.68083 | 9.69813 | 9.63824 | 9.62887 | 10.04178 |
| **IL17F** | 0.41984 | 1.32531 | 3.11670 | -2.58927 | -2.58479 | -2.27505 | -1.96019 | 0.95676 |
| **IL7** | 4.40066 | 4.80436 | 5.85322 | 0.62000 | 0.41095 | 2.38262 | 2.94677 | 2.26614 |
| **IL13** | 2.34618 | 2.19718 | 2.81776 | -0.37567 | -0.37119 | No Data | -0.35192 | -0.29686 |
| **IL18** | 11.46239 | 10.95911 | 13.21483 | 9.98085 | 9.79370 | 11.27399 | 11.50017 | 11.29079 |
| **CCL13** | 13.62575 | 11.43623 | 14.54688 | 11.91378 | 11.53247 | 12.34109 | 12.62632 | 12.23076 |
| **TNFSF10** | 10.00607 | 9.55135 | 10.14172 | 8.50483 | 9.01268 | 9.30265 | 9.50535 | 9.10515 |
| **CXCL10** | 12.13813 | 11.89799 | 14.58880 | 9.63907 | 11.49836 | 13.08127 | 10.92405 | 11.38902 |
| **IFNG** | 6.49247 | 7.81459 | 7.41790 | 2.31589 | 4.78056 | 2.89628 | 3.32296 | 3.29158 |
| **IL10** | 5.83550 | 7.79390 | 9.11201 | -1.66546 | 1.06167 | 1.20547 | -0.40727 | -0.20196 |
| **CCL19** | 12.94498 | 14.42088 | 16.73934 | 11.41022 | 13.92912 | 14.30967 | 13.89893 | 12.05047 |
| **TNF** | 5.76421 | 5.66392 | 7.93585 | 0.79959 | 1.36342 | 1.95156 | 1.24444 | 1.09342 |
| **IL15** | 7.59526 | 7.02650 | 8.49447 | 2.72473 | 2.76519 | 4.48365 | 2.89247 | 2.65174 |
| **CCL3** | 8.83422 | 8.54669 | 11.36571 | 8.22717 | 8.43223 | 8.25243 | 7.98974 | 8.66984 |
| **CXCL8** | 9.62585 | 8.42496 | 10.74287 | 7.93029 | 8.34588 | 8.49926 | 8.13117 | 8.56009 |
| **MMP12** | 9.24776 | 9.13972 | 9.27648 | 8.30618 | 8.44004 | 6.90714 | 8.06831 | 8.35793 |
| **CSF2** | 4.36333 | 4.81662 | 5.64146 | 3.92128 | 3.43852 | 3.79589 | 4.04391 | 3.50403 |
| **CSF3** | 5.47498 | 5.81935 | 5.81889 | 4.71654 | 4.58156 | 5.76869 | 4.59079 | 4.56694 |
| **VEGFA** | 13.57355 | 12.68473 | 13.78462 | 11.34496 | 11.82866 | 12.84709 | 12.77893 | 12.31737 |
| **IL17C** | 6.39959 | 7.71093 | 7.66043 | 4.60619 | 5.06725 | 6.65967 | 5.22962 | 4.18003 |
| **EGF** | 11.16666 | 9.31181 | 12.89084 | 7.64171 | 4.31002 | 9.11590 | 9.78759 | 9.76116 |
| **CCL2** | 13.62990 | 12.25407 | 15.62342 | 12.32687 | 12.71764 | 12.75407 | 12.09752 | 12.37863 |
| **IL17A** | 3.31161 | 3.73794 | 5.08035 | -3.90482 | -3.90034 | -1.41075 | -3.88107 | -3.83483 |
| **OSM** | 7.54169 | 5.46681 | 6.09144 | 0.92499 | 0.32184 | 1.52854 | 4.73856 | 4.25188 |
| **CSF1** | 11.41563 | 11.56859 | 11.98247 | 10.66121 | 11.34306 | 11.42167 | 11.35297 | 10.98836 |
| **CCL4** | 9.88183 | 9.21630 | 11.85495 | 9.48142 | 8.55183 | 9.49162 | 9.06100 | 9.82127 |
| **CXCL11** | 10.77936 | 10.60852 | 13.84528 | 8.60431 | 9.63710 | 10.94075 | 11.65568 | 10.15527 |
| **LTA** | 6.39970 | 7.25373 | 8.35147 | 4.69528 | 6.31551 | 5.62265 | 6.10181 | 5.69510 |
| **CCL7** | 5.04064 | 4.44212 | 8.67390 | 3.25835 | 6.28448 | 5.30535 | 2.92494 | 4.49792 |
| **MMP1** | 13.05088 | 12.20532 | 12.54678 | 10.32288 | 9.02290 | 12.48913 | 12.18065 | 14.47845 |

**Supplementary Table 2: Raw NPX values of cytokines**

Supplementary Table 2 presents a raw normalised protein expression (NPX) values for circulating cytokines measured in plasma samples from patients with systemic lupus erythematosus during flare and remission states. NPX values represent log_2_-scaled relative protein abundance generated using a targeted immunoassay platform.

Raw cytokine NPX values for all samples are provided in the supplementary information.

| 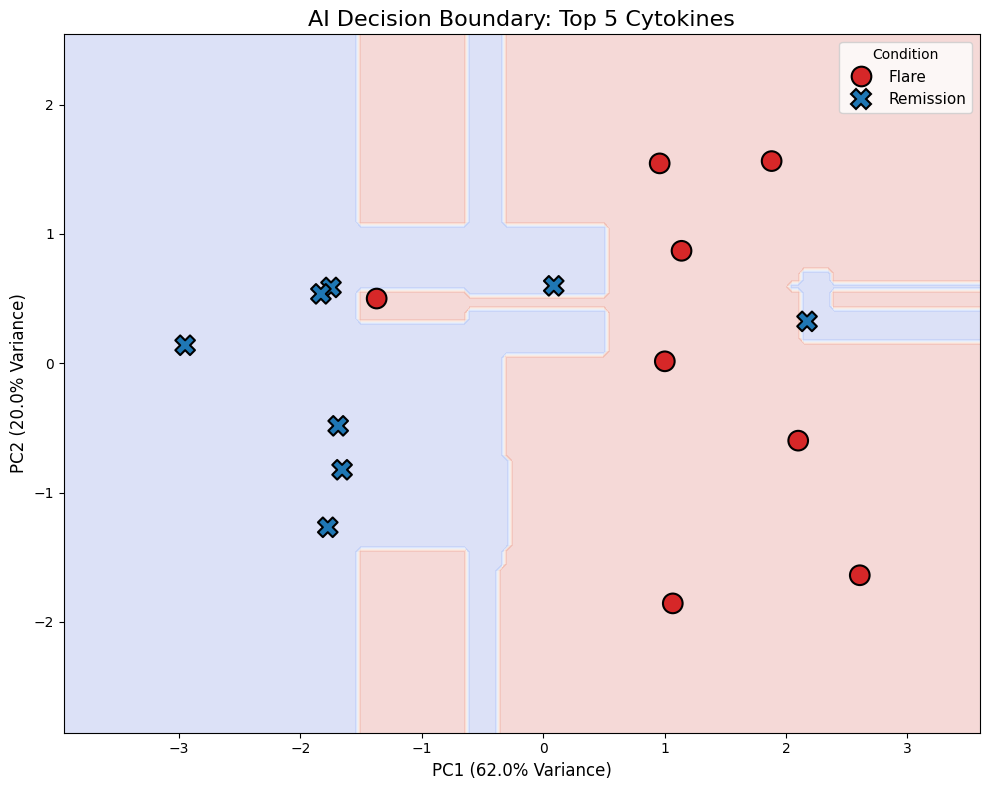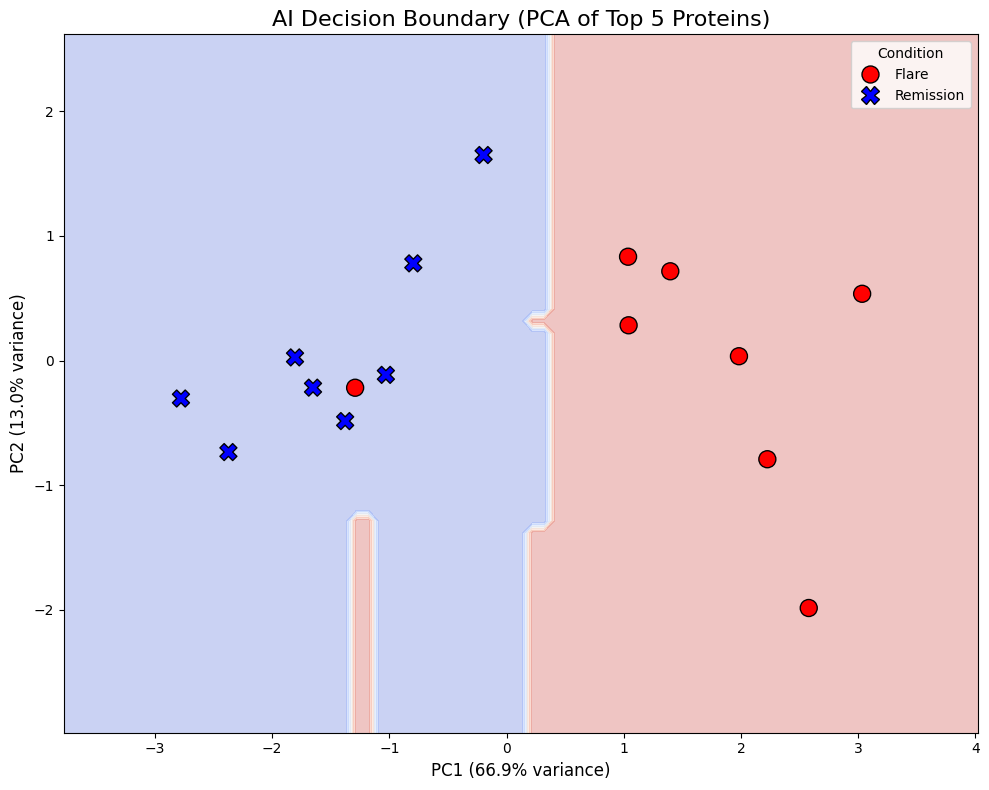  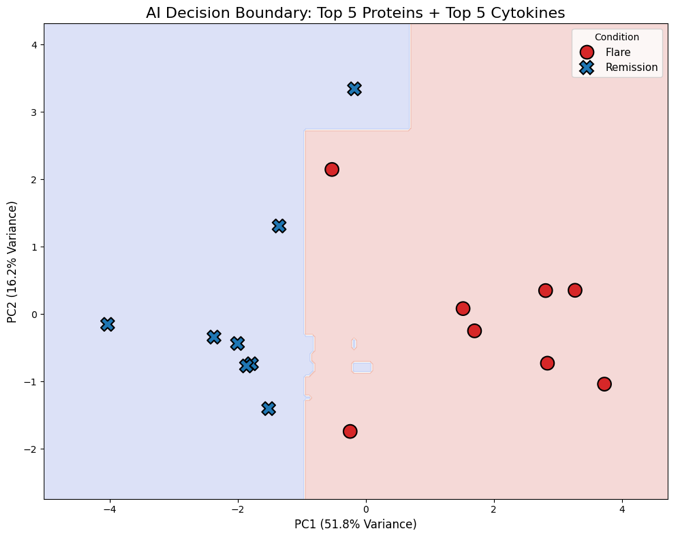 |
| --- |

**Supplementary Figure 6: AI Decision Boundaries**

**Supplementary Figure 6a: AI Decision Boundary (PCA of Top 5 Proteins).** Similar to Figure 1, this plot focuses on the top 5 proteins, with PC1 explaining 66.9% variance and PC2 13.0%. The decision boundary shows clear separation, though with slight overlap near the boundary. The inference is that protein-based biomarkers alone achieve high separation, implying they capture key variance in disease activity and could serve as a focused panel for clinical monitoring.

**Supplementary Figure 6b: AI Decision Boundary: Top 5 Cytokines.** This PCA visualization uses the top 5 cytokines, with PC1 at 62.0% variance and PC2 at 20.0%. The boundary is less linear, with some intermingling of points, indicating moderate separation. This suggests cytokines contribute to distinguishing flare from remission but may be more variable, potentially reflecting inflammatory dynamics in SLE flares.

**Supplementary Figure 6c: AI Decision Boundary: Top 5 Proteins + Top 5 Cytokines.** Combining proteins and cytokines, PC1 explains 51.8% variance and PC2 16.2%. The plot demonstrates improved clustering compared to individual sets, with tighter groups and minimal overlap. This infers synergistic effects between proteins and cytokines, enhancing overall model accuracy for disease state classification

| 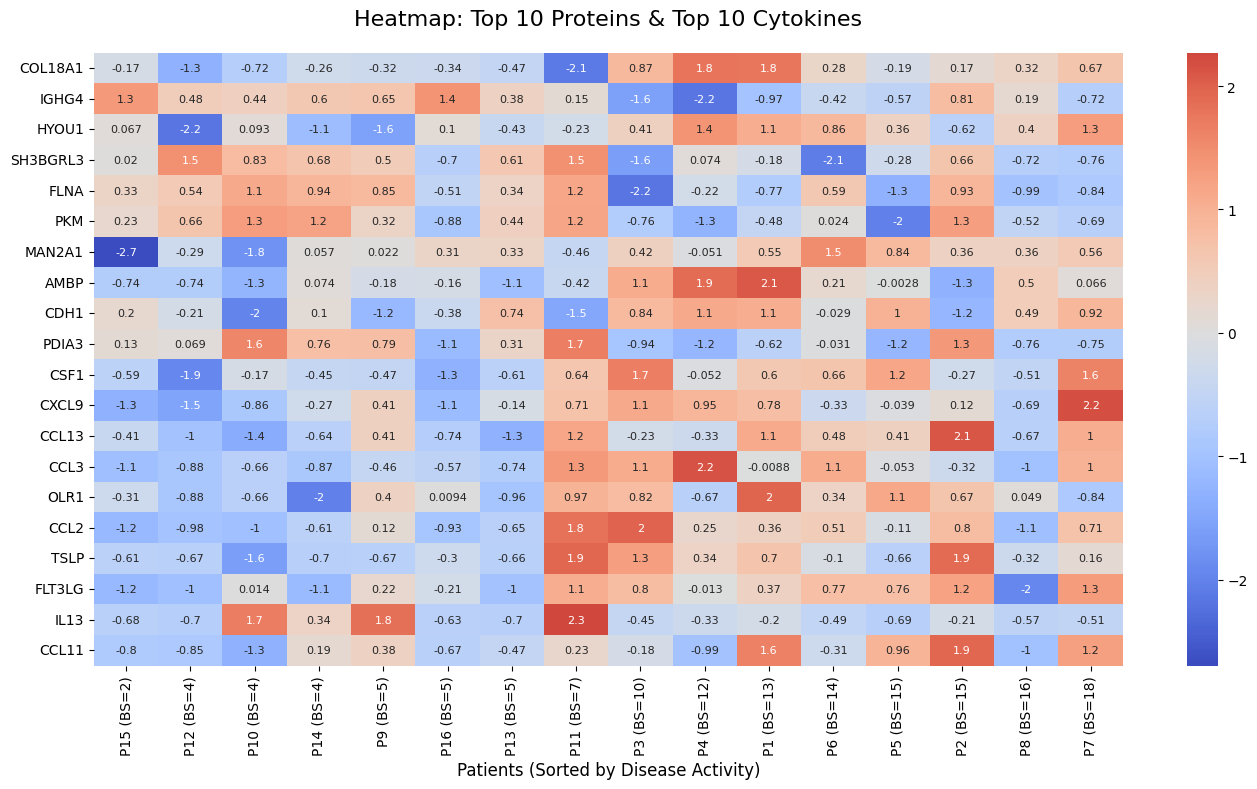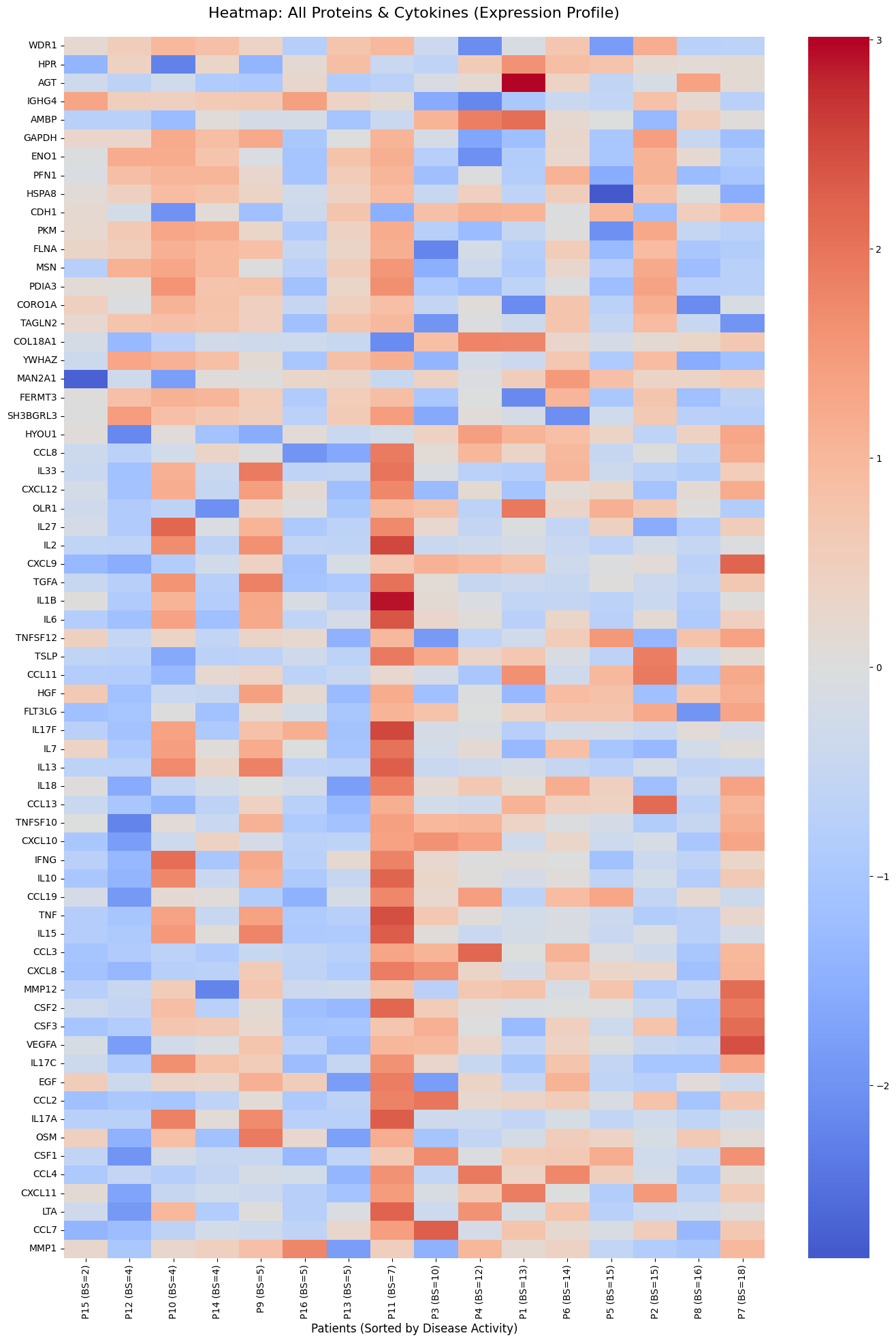 |
| --- |

**Supplementary Figure 7: Heatmaps**

**Supplementary Figure 7a: Heatmap: Top 10 Proteins & Top 10 Cytokines.** The heatmap displays expression levels across patients sorted by disease activity, with a color scale from blue (low) to red (high). Patterns show upregulation in flare patients for markers like COL18A1 and CCL11. This signifies differential expression correlated with activity, implying these biomarkers track progression and could inform personalized monitoring.

**Supplementary Figure 7b: Heatmap: All Proteins & Cytokines (Expression Profile).** Extending Figure 9, this comprehensive heatmap includes all measured molecules, revealing broader expression clusters. Distinct blocks of up- and downregulation align with disease activity gradients. The inference is a systemic shift in the proteome and cytokine profile during flares, highlighting potential multi-marker signatures for SLE.

### **Discussion**

This exploratory proteomic analysis highlights vascular stress and cytoskeletal integrity as polarized molecular themes in lupus flare versus remission. While limited by sample size, consistent associations of COL18A1, AGT, FLNA, and TAGLN2 underscore their potential as dynamic biomarkers. Cytokine profiling further identified reproducible flare-associated signals (CXCL10, IL18, VEGFA), providing interpretable anchors within complex datasets. Integration of STRING and Reactome outputs into multi-omics pipelines and real-world datasets may enhance biomarker discovery and precision stratification. Together, these findings support a precision medicine framework where molecular signals of vascular stress and cytoskeletal breakdown provide objective, organ-level insights into disease activity. Future validation in larger, diverse cohorts will be essential to translate these exploratory signals into clinically actionable biomarkers. Integration of these exploratory proteomic signals into multi-omics pipelines and real-world datasets will be essential to validate their utility for precision stratification in lupus.
